# Target gene responses differ when transcription factor levels are acutely decreased by nuclear export versus degradation

**DOI:** 10.1242/dev.202775

**Published:** 2024-11-08

**Authors:** James McGehee, Angelike Stathopoulos

**Affiliations:** California Institute of Technology, Division of Biology and Biological Engineering, 1200 East California Boulevard, Pasadena, CA 91125, USA

**Keywords:** Dorsal transcription factor, *Drosophila melanogaster*, LEXY and B-LID, Dorsal-ventral patterning, Gene expression dynamics, Optogenetics

## Abstract

Defining the time of action for morphogens requires tools capable of temporally controlled perturbations. To study how the transcription factor Dorsal affects patterning of the *Drosophila* embryonic dorsal-ventral axis, we used two light-inducible tags that trigger either nuclear export or degradation of Dorsal under blue light. Nuclear export of Dorsal leads to loss of the high-threshold, ventrally expressed target gene *snail* (*sna*), while the low-threshold, laterally expressed target gene *short-gastrulation* (*sog*) is retained. In contrast, degradation of Dorsal results in retention of *sna*, loss of *sog*, and lower nuclear levels compared to when Dorsal is exported from the nucleus. To understand why nuclear export causes loss of *sna* but degradation does not, we investigated Dorsal kinetics using photobleaching and found that it rapidly re-enters the nucleus even under blue-light conditions favoring export. The associated kinetics of Dorsal being rapidly imported and exported continuously are likely responsible for loss of *sna* but, alternatively, can support *sog*. Collectively, our results indicate that this dynamic patterning process is influenced by both Dorsal concentration and nuclear retention.

## Teaser

**Summary:** Optogenetic tools can be used to determine how transcription factor levels and nuclear retention impact a dynamic patterning process.

## INTRODUCTION

Morphogens are proteins that form concentration gradients in developing organisms to regulate the expression of target genes (Kicheva and Briscoe, 2023; Rogers and Schier, 2011). Key morphogens include signaling molecules like sonic hedgehog (SHH) and bone morphogenic protein (BMP), as well as transcription factors (TFs) such as Bicoid and Dorsal (DL) (Briscoe and Small, 2015; Greenfeld et al., 2021). While the spatial regulation of morphogen gradients is well understood, investigating their time of action has been more challenging (Kutejova et al., 2009; Nahmad and Lander, 2011; Rushlow and Shvartsman, 2012). Some studies indicate that morphogen signals can be integrated over time to influence target gene expression, highlighting the importance of signal duration (Sagner and Briscoe, 2017; Huang et al., 2017). However, determining the temporal role of a morphogen using genetic knockouts and epistasis can be difficult, as multiple roles may complicate the gene regulatory network progression. Techniques that allow for precise spatiotemporal control of protein levels are therefore essential. Optogenetics enables the control of genetically encoded proteins with light and has proven valuable for studying morphogen patterning (Rogers et al., 2020; Huang et al., 2017; McDaniel et al., 2019; Singh et al., 2022; Johnson et al., 2017). In this study, we have used two optogenetic tags – blue light inducible degradation domain (BLID; Bonger et al., 2014) and a light inducible nuclear export system (LEXY; Niopek et al., 2016) – to assess how different perturbations to nuclear DL levels affect target gene expression during *Drosophila melanogaster* embryogenesis.

The early stages of *Drosophila* embryogenesis consist of rapid DNA replication and nuclear divisions called nuclear cycles. Many zygotic genes are activated during nuclear cycle (nc) 14, which is the longest nc of early embryogenesis. During this time, DL forms a nuclear concentration gradient across the dorsal-ventral (DV) axis leading to expression of high threshold target genes in ventral regions and low threshold target genes more dorsally in ventrolateral and lateral regions (Reeves and Stathopoulos, 2009). While DL levels are instrumental in specifying target gene expression in a spatially-instructive manner consistent with morphogen outputs, nuclear DL levels are also dynamic and increase during these early stages, which raises the questions when and for how long does DL concentration have an effect on target gene expression (Rushlow and Shvartsman, 2012).

In a previous study, we used BLID to degrade DL at certain points in time to understand how the loss of nuclear DL during certain nuclear cycles affects target gene expression (Irizarry et al., 2020). We found that *snail* (*sna*), which is thought to require high nuclear DL levels, can be activated independently of peak DL levels achieved in late nc14. This suggests that DL input is necessary earlier, but once the DL-target gene *twist* (*twi*) is expressed, DL is no longer required. Supporting this model, we discovered that the late activation of *sna* depends on the Twi TF. Although BLID allows for spatiotemporal control of TF removal under blue light, the reversibility of the system relies on nascent protein synthesis occurring after the light is removed. For DL, no nascent DL-BLID is produced during these embryonic stages. Therefore, while DL-BLID can indicate when DL is no longer needed for *sna* expression (i.e. late 14), it does not allow us to distinguish the early temporal role of DL from its later role.

To investigate this, the LEXY sequence (Niopek et al., 2016; Kögler et al., 2021; Singh et al., 2022; Zhao et al., 2023 preprint) was added to DL, enabling reversible nuclear protein depletion through blue-light inducible nuclear export. The effects of DL depletion were investigated by live imaging target gene expression using the MS2/MCP system, which detects RNA stem-loops in nascent transcripts (Garcia et al., 2013a; Lucas et al., 2013). By combining BLID, LEXY and live imaging, we found that the *sna* regulatory system responds differently to nuclear DL loss when comparing DL-LEXY to DL-BLID. Although DL-LEXY is associated with higher nuclear DL levels than DL-BLID, *sna* is lost with LEXY but retained with BLID under blue light. This difference indicates that import-export kinetics influence target gene expression and are crucial regulatory factors, alongside concentration, for patterning the DV axis.

## RESULTS

### DL is exported from the nucleus under blue light in *dl-LEXY*

**Fig. 1.**
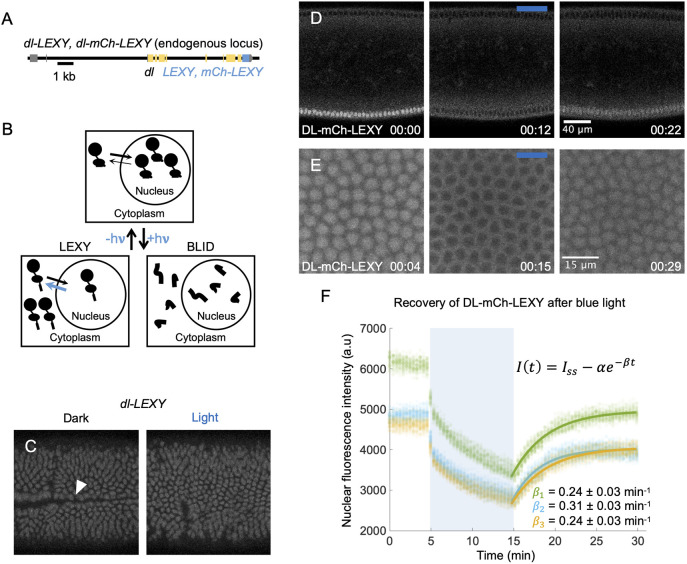
**Dorsal is exported from the nucleus under blue light in *dl-LEXY*.** (A) A schematic of the *dl-LEXY* and *dl-mCh-LEXY* CRISPR constructs. (B) Blue light reveals a NES in LEXY, leading to nuclear export, or a degron in BLID, leading to degradation. (C) Stills of gastrulation succeeding in the dark and failing under blue light in *dl-LEXY*. The white triangle marks the furrow. Of control embryos with no DL-LEXY, 3/4 undergo gastrulation under blue light, 6/6 embryos for *dl-LEXY* undergo gastrulation in the dark, and 2/9 embryos for *dl-LEXY* undergo gastrulation under blue light. (D) Stills during nc14 from a movie of DL-mCh-LEXY before (00:00), during (00:12) and after (00:22) blue light exposure. Time stamps represent the amount of time since image acquisition began. The blue bar represents images taken under blue light. (E) Magnified views of DL-mCh-LEXY before (00:04), during (00:14) and after (00:29) blue light exposure. (F) Quantification of the nuclear levels of DL-mCh-LEXY in E. Nuclei were tracked over time and the recovery after the removal of blue light for each individual nucleus was fit to a single exponential shown in F. The average parameters for this fit were calculated and used to display the fit in F (solid colored lines). Each color corresponds to a single embryo (the number of nuclei for embryo 1 *n*=52, for embryo 2 *n*=51 and for embryo 3 *n*=47). Embryos in C and E are oriented with the anterior to the left, posterior to the right and ventral facing out of the page. The embryo in D is oriented with the same anterior and posterior position, but with dorsal at the top and ventral at the bottom. The embryo in D was collected from a mixed cage of homozygous and heterozygous mothers and embryo in E was collected from a cage of homozygous mothers, but both are from the same stock.

To develop a system where we could control nuclear DL levels reversibly, we used CRISPR/Cas9 genome editing to construct in-frame fusions of DL and DL-mCherry to the LEXY tag (Niopek et al., 2016), generating the *Drosophila* stocks *dl-LEXY* and *dl-mCh-LEXY* (Fig. 1A,B, ‘LEXY’). To determine whether DL-LEXY and DL-BLID have an effect on target gene expression in the dark, embryos were stained for *sna* and the normalized widths (i.e. width divided by the individual embryo circumference) were computed. Although the mean *sna* expression domain width was slightly narrower in *dl-LEXY* and *dl-BLID* compared to control, these differences were not significant and suggest the fusions are similarly functional (Fig. S1A,C; Bothma et al., 2015; Irizarry et al., 2020). We also compared the DL gradients in *dl-LEXY* and *dl-BLID* with control (*yw*) to determine whether the gradients were strongly affected by addition of LEXY or BLID in the dark (Fig. S1B). When the DL gradient was fitted to a Gaussian function (Fig. S1D,E; Trisnadi et al., 2013), the peak levels of DL and scaling factor (σ) in *dl-LEXY* and control were similar (Fig. S1F,G). In *dl-BLID*, the levels were reduced and there was also a change in the scaling factor (σ) (Fig. S1F,G). One caveat of this fixed data is that embryos were exposed to ambient light during processing, which may have a greater effect on DL-BLID. While *dl-LEXY* and *dl-BLID* both supported relatively normal *sna* expression, the DL gradient in *dl-LEXY* was more similar to that in control, suggesting that the *dl-LEXY* background may be more useful for analysis of dynamic requirements for DL in supporting target gene expression.

When embryos with DL-LEXY were treated with blue light, it resulted in export of the fusion protein from the nucleus as detected by following the mCherry signal associated with DL-mCh-LEXY during live imaging (Fig. 1D,E; Movies 1,2). The levels of DL were quantified and tracked over time for each individual nucleus in three embryos (Fig. 1E,F; Movie 2). After the blue light was removed, DL-mCh-LEXY again entered nuclei with an inverse time scale of 0.24±0.03 min^−1^, 0.31±0.03 min^−1^ and 0.24±0.03 min^−1^ for three individual embryos averaged across nuclei. This process of export and reimport can be repeated multiple times (Movie 1), which makes it possible to use live imaging to test how temporarily removing DL affects target gene expression.

To determine whether loss of nuclear DL-LEXY through blue light illumination has a phenotypic effect, embryos were kept in the dark or exposed to blue light and imaged to determine if gastrulation occurred. Under blue light, three of four control embryos (with no DL-LEXY) gastrulated, whereas six of six embryos gastrulated in the dark for the homozygous *dl-LEXY* stock. This suggests that blue light leads to some lethality. However, when the *dl-LEXY* stock was exposed to blue light, only two of nine embryos gastrulated. These results demonstrate that loss of nuclear DL results in embryos that fail to undergo gastrulation, which is lethal (Fig. 1C). With this knowledge, we sought to test how two different approaches to perturb DL levels, transiently with DL-LEXY or permanently with DL-BLID, affect gene expression.

### Sites of active *sna* transcription are lost in *dL-LEXY* but recover in *dL-BLID* when DL is removed with blue light at mid-nc14

**Fig. 2.**
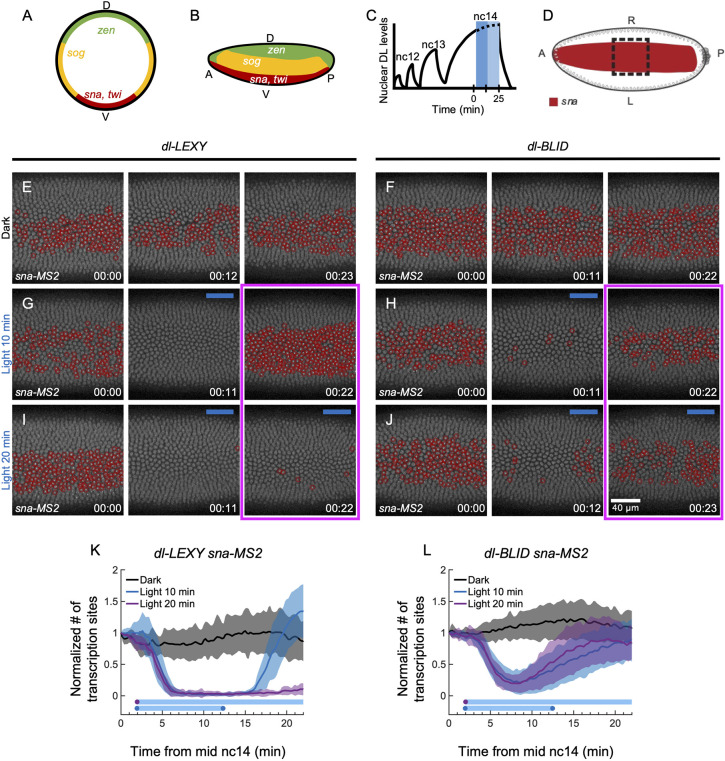
**Sites of active *sna* transcription are lost in *dL-LEXY* but recover in *dL-BLID* when DL is removed with blue light at mid-nc14.** (A,B) Expression of DL target genes along the DV axis in a cross section (A) or lateral view (B) with high threshold targets (*sna* and *twi*; red), low threshold targets (*sog*; yellow) and repressed targets (*zen*; green). (C) Illumination scheme relative to schematic of nuclear DL concentration trends over time from previously quantified data (Reeves et al., 2012). Embryos were illuminated for 10 min (dark blue shaded region) or 20 min (dark blue plus light blue shaded region) with blue light during mid-nc14. (D) A schematic of the *sna* domain (red) in a ventral view. The black dashed box represents the field of view and the area of blue light illumination. (E,G,I) *sna-MS2* expression in *dl-LEXY* during late nc14, when kept in the dark (E), illuminated for 10 min (G) or illuminated for 20 min (I), at 0 min (00:00), 11 min (00:11 and 00:12) and 22 min (00:22 and 00:23). (F,H,J) Similar conditions to E,G,I, except for *dl-BLID*. (K,L) The normalized mean number of foci, or transcription sites (mean±s.d., *n*=6 for each, except for dl-LEXY light 20 min, *n*=7), detected for *sna-MS2* in *dl-LEXY* (E,G,I) and *dl-BLID* (F,H,J) at each time point. This quantification is not cumulative and provides the instantaneous number of transcription sites. For each embryo, the mean number of transcription sites was divided by the starting number to normalize the data. Black is in the dark, blue is 10 min illumination and purple is 20 min illumination. Embryo ventral views are shown in E-J: anterior to the left, posterior to the right, and ventral facing out of the page, with field of view positioned in the center of the trunk. Foci are circled in red for *sna*. *t*=0 indicates the start of imaging, at mid-nc14, as determined by the cellularization front having progressed 50% of nuclei length. In this and all other figures, blue bars represent frames under blue light and time stamps are h:min. Embryos of a certain genotype were collected from the same cage either on the same day or subsequent days.

DL activates expression of an array of target genes including the high threshold target *sna* in the ventral region (Fig. 2A,B). To test the effect of removing DL transiently at mid-nc14, when nuclear concentration of DL in ventrally-positioned nuclei is highest (Reeves et al., 2012), embryos from *dl-LEXY* mothers were illuminated with blue light for 10 min or 20 min and compared to embryos that were kept in the dark during a similar period of time (Fig. 2C). To monitor expression of *sna*, we used a previously published MS2 reporter that was inserted at an exogenous location (Fig. S2A; Bothma et al., 2015; Irizarry et al., 2020). Using the MS2-MCP imaging system, we were able to observe expression of *sna* over time from a ventral viewpoint (Fig. 2D). We previously found that *sna* transcription at later stages of nc14 can be independent of high levels of DL, as nascent transcription is detected in embryos laid by *dl-BLID* mothers that develop under blue light at mid/late-nc14 (Irizarry et al., 2020). To compare *dl-LEXY* and *dl-BLID*, we reimaged *dl-BLID* under the same conditions as *dl-LEXY.*

In the dark, sites of active transcription were detected continuously for *sna* in ventral nuclei during mid-nc14 in *dl-LEXY* (Fig. 2E; Movie 3) and *dl-BLID* (Fig. 2F; Movie 3). After 10 min of blue light exposure, sites of nascent *sna* transcription were undetectable in most nuclei in *dl-LEXY* (Fig. 2G 00:11; Movie 3) or were detected in only a few nuclei in *dl-BLID* (Fig. 2H 00:11; Movie 3). In both *dl-LEXY* and *dl-BLID*, sites of active *sna* transcription were detected after returning to the dark (Fig. 2G,H 00:22; Movie 3). Under 20 min of blue light exposure, sites of nascent *sna* transcription remained undetectable in *dl-LEXY* (Fig. 2I 00:11 and 00:22; Movie 3). In contrast, when *dl-BLID* was exposed to 20 min of blue light, the number of active *sna* transcription sites initially decreased before increasing (Fig. 2J 00:12 and 00:23; Movie 3), similar to the case of 10 min of blue light (Fig. 2H). These results agree with our previous study for *dl-BLID* in that *sna* expression is supported despite prolonged blue light illumination (Irizarry et al., 2020), and highlight that *dl-LEXY* results in a different outcome. Specifically, these data demonstrate that nuclear DL is required to maintain sites of active *sna* transcription at mid/late-nc14 in *dl-LEXY* but not in *dl-BLID*.

To support these experiments, at least six movies were obtained for each of the three conditions: dark, 10 min blue light, and 20 min blue light, for both *dl-LEXY* and *dl-BLID*. To quantify the changes in the number of active transcription sites, foci were detected and counted over time, and normalized by the starting number of active sites. Upon blue light illumination, the quantification shows the initial decrease in the number of active transcription sites in both *dl-LEXY* and *dl-BLID* (Fig. 2K,L; Fig. S2C,D), and how this number increases at later time points when blue light remains on in *dl-BLID*, but not in *dl-LEXY* (Fig. 2K,L, ‘Light 20 min’; Fig. S2C,D). The number of active *sna* transcription sites in *dl-BLID* increases after ∼10 min, regardless of whether the embryo was exposed to blue light for 10 min or 20 min (Fig. 2L; Fig. S2D). Since the starting levels of DL vary and could potentially affect the expression of target genes, we compared the mean starting number of transcription sites between *dl-BLID* and *dl-LEXY* to determine if there was a statistically significant difference. This is possible because no light has been applied during the first time point of the movies. We found no significant difference in the mean number of starting *sna* transcription sites for *dl-LEXY* and *dl-BLID* before blue light was applied (Fig. S2B), demonstrating that the starting number of transcription sites does not explain the difference we see between *dl-BLID* and *dl-LEXY*. The variability likely relates to stochastic uncaging of the optogenetic tags. Importantly, these results demonstrate that blue light illumination results in different trends in *sna* transcription for *dl-BLID* versus *dl-LEXY*, showing that transcription can recover when DL is degraded (BLID) but not when it is exported (LEXY) (Fig. 2K,L; Fig. S2C,D).

### In *dl-LEXY*, *sog* expression is supported even under blue light

**Fig. 3.**
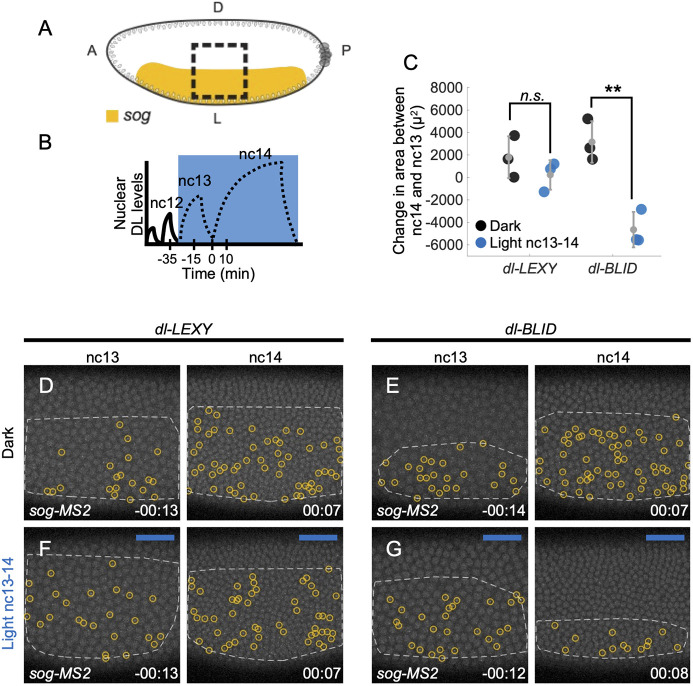
**In *dl-LEXY*, *sog* expression is supported even under blue light.** (A) A schematic of the *sog* domain (yellow) transcription in a dorsal-lateral view. The dashed black box represents the field of view and the area of blue light illumination. (B) Illumination scheme displayed relative to nuclear DL concentration trends over time (schematic). Embryos were illuminated with continuous blue light in nc13 through nc14. (C) Quantification of the change in area between nc14 and nc13 for *sog-MS2* in *dl-LEXY* and *dl-BLID*. Black markers represent the dark and blue markers represent illumination from nc13-14 (in gray, mean±s.d., *n*=3 for each). When comparing dark to light at nc13-14, only *dL-BLID* is significantly different, *P*=0.002 (Tukey's HSD for multiple comparisons after performing one way ANOVA). (D,F) *sog-MS2* in *dl-LEXY* when kept in the dark (D) or when illuminated continuously from nc13 through nc14 (F) at nc13 (−00:13) and early-nc14 (00:07). (E,G) Similar to D,F, except for *dl-BLID*. Foci are circled in yellow for *sog*. *t*=0 indicates the start of nc14. Dorsal-lateral views are shown. Embryos of a certain genotype were collected from the same cage either on the same day or subsequent days. White dashed lines indicate the area of cumulative expression that is observable in the field of view for the corresponding nuclear cycle.

As *dl-LEXY* and *dl-BLID* result in a different number of active *sna* transcription sites after blue light illumination (Fig. 2), we sought to determine whether the phenomenon was unique to *sna*, or if other targets of DL behaved similarly. Another target gene is *short gastrulation* (*sog*), which is a low threshold target and is expressed broadly in lateral regions (reviewed by Reeves and Stathopoulos, 2009). Sna acts as a transcriptional repressor to repress *sog* expression in the ventral domain, refining *sog* expression to two lateral stripes. DL also acts directly as a repressor to limit the expression of the gene *zerknüllt* (*zen*) to dorsal regions. Expression of *zen* is thought to respond to a similar threshold as *sog*, which results in *zen* having an inverse pattern compared to *sog* (Fig. 2A,B). To test the effects of changing nuclear DL levels using *dl-LEXY* and *dl-BLID* on these target genes, we created *sog-MS2* and *zen-MS2* at their endogenous loci using CRISPR/Cas9 (Fig. S2A). Previously, changes in the *sog* boundary in fixed samples were only detected if we illuminated *dl-BLID* embryos for a prolonged period (Irizarry et al., 2020). Thus, we illuminated continuously from the end of nc12 until late nc14 when germ-band extension was observed (Fig. 3B).

To detect differences in the *sog* and *zen* expression domains, we imaged in a dorsal-lateral field of view, which captures the dorsally-positioned boundary of *sog* and the boundary of *zen* and quantified the change in domain (i.e. area of expression) between nc13 and nc14 (Fig. 3A,C). For *sog-MS2*, signal was detected in the dark at nc13 and nc14 in *dl-LEXY* (Fig. 3D; Movie 4) and *dl-BLID* (Fig. 3E; Movie 4). When illuminated during nc13-14, there was not a significant difference in the position of the dorsal boundary of the *sog* domain in *dl-LEXY* (Fig. 3C,F; Movie 4), but there was a ventral retraction of the *sog* boundary leading to fewer detected sites of transcription in the field of view in *dl-BLID* (Fig. 3C,G; Movie 4). In contrast to *sog*, the *zen* boundary position remained unchanged by any of these similar perturbations (Fig. S3; Movie 7; see Discussion).

### Under blue light, DL levels are equal or higher in *dl-LEXY* compared to *dl-BLID*

**Fig. 4.**
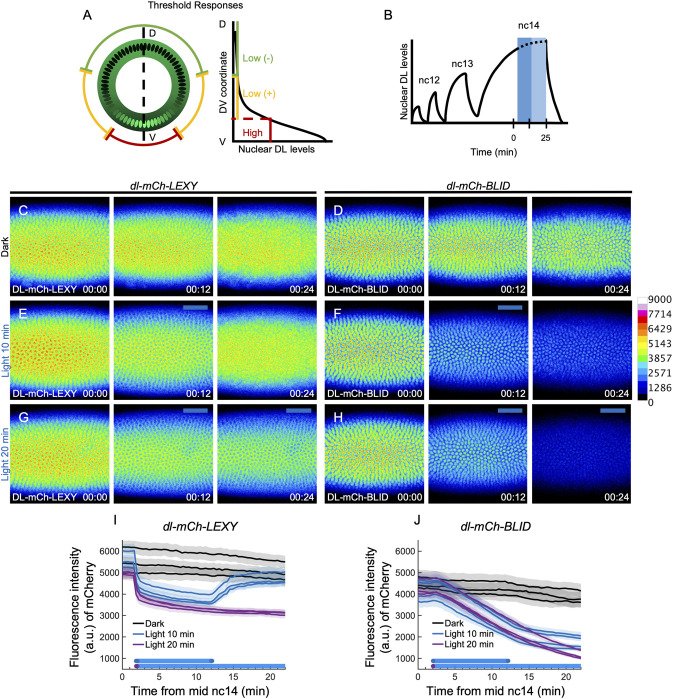
**Under blue light, DL levels are equal or higher in *dl-LEXY* compared to *dl-BLID*.** (A) A model of threshold responses to the DL nucleo-cytoplasmic gradient (green, inner circle) shown in cross-section embryo view where the highest levels of nuclear DL correspond to ventral genes (red) and lower nuclear DL levels correspond to lateral genes (yellow). DL also represses genes that should be expressed dorsally (green, outer circle). (B) Illumination scheme: embryos were illuminated with 10 min (dark blue shaded region) or 20 min (dark blue plus light blue shaded region) of blue light in mid-nc14. (C,E,G) Stills from live imaging of DL-mCh-LEXY during late nc14, when kept in the dark (C), illuminated for 10 min (E) or illuminated for 20 min (G), at 0 min (00:00), 12 min (00:12), and 24 min (00:24). (D,F,H) Similar conditions to C,E,G, except for DL-mCh-BLID. (I,J) Plots of the mean DL-mCh-LEXY (I) or DL-mCh-BLID (J) fluorescence intensity (mean±s.d., *n*=3 for each except for DL-mCh-BLID 10 min light where *n*=4) for each embryo assayed. Black lines are individual embryos kept in the dark, blue lines are individual embryos with 10 min of light, and purple lines are individual embryos with 20 min of light. Images C-H are displayed using a rainbow colormap, where high intensity pixels are in white and pink, and low intensity pixels are in blue and black. Embryo ventral views are shown with the field of view positioned in the center of the trunk. *t*=0 indicates the start of imaging, at mid-nc14, as determined by the cellularization front having progressed 50% of nuclei length. Embryos of a certain genotype were collected from the same cage either on the same day or subsequent days.

To potentially explain the differences we observed in *dl-LEXY* and *dl-BLID* for *sna* and *sog*, we assayed the levels of nuclear DL in these lines under blue light. In the traditional threshold model, *sna* expression requires the highest levels of DL (Fig. 4A). In this model, we would predict that DL levels are higher in *dl-BLID* than in *dl-LEXY* when under blue light. To test this, embryos laid by *dl-mCh-LEXY* and *dl-mCh-BLID* mothers were illuminated with 10 min and 20 min of blue light (Fig. 4B), as done in the previous *sna* experiment (Fig. 2). In the dark for both *dl-mCh-LEXY* and *dl-mCh-BLID*, there was continuous nuclear DL present (Fig. 4C,D,I,J ‘Dark’; Movie 5). Upon 10 min of blue light illumination, DL-mCh-LEXY was exported out of the nucleus, resulting in a decrease in nuclear DL levels and an increase in cytoplasmic DL levels (Fig. 4E,I 00:12; Movie 5). Upon returning to the dark, nuclear DL levels began to increase. Nuclear DL levels in the dark after being illuminated for 10 min were similar to the nuclear DL levels of embryos kept in constant darkness (Fig. 4E 00:24 compare with Fig. 4C 00:24 and quantified in Fig. 4I; Movie 5). On the other hand, DL-mCh-BLID levels decreased in both the cytoplasm and the nucleus under 10 min blue light (Fig. 4F,J 00:12). This trend continued even after the blue light was removed, suggesting that there is a slight delay in ending the degradation of DL (Fig. 4F,J 00:24; Movie 5). Upon 20 min of blue light illumination, DL-mCh-LEXY stayed cytoplasmic and maintained low levels of nuclear DL (Fig. 4G,I; Movie 5), whereas DL-mCh-BLID levels decreased even more than the 10 min exposure (Fig. 4H,J; Movie 5). This is expected, as the longer the degron is uncaged, the more degradation of DL should occur, resulting in lower nuclear levels.

These data show that the resulting levels of nuclear DL in DL-mCh-BLID were lower than the levels in DL-mCh-LEXY when both were under 20 min of blue light. Similarly, the levels of nuclear DL under 10 min of blue light were higher in *dl-LEXY* than *dl-BLID*. While this difference in nuclear DL levels can explain the differences in *sog* expression (i.e. only *dl-BLID* but not *dl-LEXY* levels may fall below the threshold required to support *sog*), it fails to explain the difference in the number of *sna* transcription sites observed under 20 min of blue light. Since DL is a positive input to *sna*, it would be expected that *sna* expression would occur where DL levels are higher, which would be in *dl-LEXY*. However, we found that there are a greater number of *sna* transcription sites in *dl-BLID* where DL levels are lower.

### Twi levels do not change at late nc14 under blue light in *dl-LEXY* or *dl-BLID*

As nuclear DL levels cannot explain all the differences we see between *dl-LEXY* and *dl-BLID*, another possible explanation is that blue light-induced export of DL may have an effect on Twi protein levels. We have previously shown that Twi is responsible for DL-independent activation of *sna* at late nc14 in *dl-BLID* under blue light. As long as Twi is produced, then DL input can be lost and *sna* expression is retained (Irizarry et al., 2020). To test whether Twi levels are differentially affected in *dl-LEXY* versus *dl-BLID* under 20 min blue light and possibly account for differences observed in *sna* (Fig. 2), we imaged Twi using a previously published Twi-LlamaTag fly stock (Bothma et al., 2018). Twi-LlamaTag allows detection of Twi protein localization live *in vivo* when maternally deposited mCherry is available. We found that the levels of Twi did not change upon 20 min blue light illumination and appeared to be similar between *dl-LEXY* and *dl-BLID* when assaying Twi-LlamaTag (Fig. S4).

An alternative explanation for how DL-LEXY and DL-BLID differentially affect *sna* expression is that DL-LEXY sequesters potential cofactors in the cytoplasm under blue light. Implicitly, this means that when DL is exported from the nucleus, any transcription factors or cofactors bound to DL would also shuttle into the cytoplasm. There is some evidence that DL and Twi might physically interact (Shirokawa and Courey, 1997). If Twi was exported with DL under blue light, a change in localization of the Twi protein using the Twi-LlamaTag should be detected; however, as stated above, we did not observe changes in Twi levels under blue light in *dl-LEXY* (Fig. S4C; Movie 8). This demonstrates that DL nuclear export does not result in an appreciable reduction of nuclear Twi.

While Twi is not exported with DL, this does not rule out that other potential cofactors might be exported with DL in *dl-LEXY* under blue light. For example, since Twi is required for *sna* transcription in *dl-BLID* under blue light, it is possible that DL and Twi share a cofactor, and it is this cofactor that is sequestered with DL in the cytoplasm in *dl-LEXY* under blue light. However, it would be difficult to test the protein localization of every DL cofactor individually, especially if the cofactor in question is unknown. Thus, we sought to explore and rule out other possible explanations for how *dl-LEXY* and *dl-BLID* result in differences in the number of *sna* transcription sites.

### FRAP demonstrates that DL-LEXY rapidly transits in and out of the nucleus, even under blue light illumination

Another model that could explain the differences between *dl-LEXY* and *dl-BLID* relates to the dynamics of how nuclear protein levels decrease with these perturbations. DL-LEXY is likely rapidly transiting in and out of the nucleus under blue light, and while degradation of DL-BLID does not change its localization, both cytoplasmic and nuclear levels decrease. When DL-LEXY is exposed to blue light, the normally-buried nuclear export sequence (NES) is revealed as the Jα helix is unfolding (Niopek et al., 2016). However, the nuclear localization sequence (NLS) of DL should be unaffected. Thus, DL would be rapidly transiting between the cytoplasm and the nucleus, due to its own NLS and a strong NES being present. Since DL is predominantly cytoplasmic under blue light, the export rate is likely much higher than the import rate, due to the strong nature of the exposed NES contained in LEXY. We hypothesized that this rapid import and export may act to disrupt DL-independent activation of *sna*. Specifically, DL might act transiently at the *sna* locus before being exported, causing a disruption in *sna* activation through other factors, such as Twi. This model suggests that *sna* needs DL to remain in the nucleus for a sustained amount of time.

**Fig. 5.**
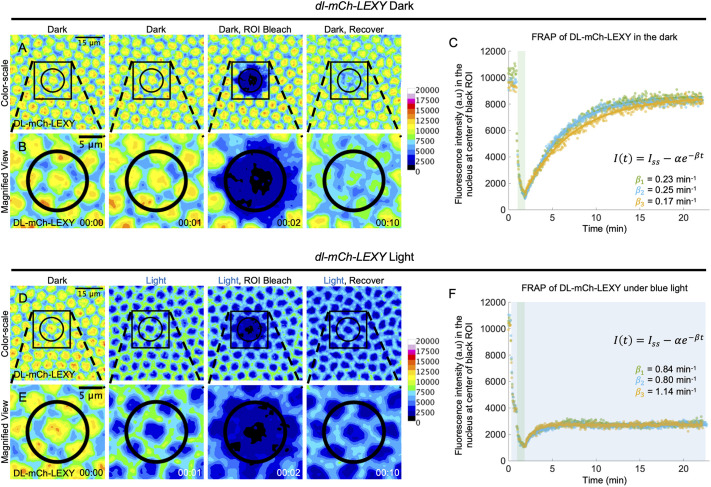
**FRAP demonstrates DL-LEXY rapidly transits in and out of the nucleus, even under blue light illumination.** (A) Stills of an embryo that underwent FRAP in the dark (Dark), after bleaching (Dark, ROI Bleach) and after recovery (Dark, Recover). Images were false-colored using a rainbow colormap, and a Gaussian blur (r=4) was performed for display. The black circular ROI marks the region that was bleached, and the black square represents the area of the magnified view in B. (B) The same as in panel A except a magnified view. (C) Plots of the mean fluorescent intensity for the nucleus at the center of the black circular ROI. The different line and marker colors represent FRAP performed on three different embryos. The green shaded region marks time points when the DL-mCh-LEXY signal was bleached (∼1 min). The recovery after bleaching was fit to the displayed equation and estimates of β, which relates to the inverse of the recovery time, are displayed for each of three embryos. Estimates of *I_ss_*, which relates to the final steady state fluorescence levels, are 8628 a.u., 8337 a.u. and 8537 a.u. Estimates of α, which relates to the difference between the final steady state fluorescence levels and the fluorescence levels at the beginning of the recovery, are 7807 a.u., 7567 a.u., and 7238 a.u. (D) A similar experimental setup to A with different embryos, where only the first panel is in the dark and all other panels are with blue light applied. (E) The same as in panel D except a magnified view. (F) The same as C, except for embryos that underwent bleaching while blue light was applied. β estimates for each of three embryos are displayed. Estimates of *I_ss_* are 2781 a.u., 2702 a.u. and 2727 a.u. Estimates of α are 1622 a.u., 1574 a.u. and 1639 a.u. The mean β is significantly different between the dark and the light (*P*=0.003, two-sample unpaired *t*-test). Ventral views of embryos are shown with the field of view positioned in the center of the trunk around the ventral-most point. *t*=0 indicates the start of imaging, at mid-nc14, as determined by the cellularization front having progressed to 50% of nuclei length.

To test whether DL is transiting in and out of the nucleus rapidly, we performed fluorescence recovery after photobleaching (FRAP; Koulouras et al., 2018; Williamson et al., 2021; Bulinski et al., 2001; Cardarelli et al., 2009) on DL-mCh-LEXY in the dark and under blue light (Movie 6). Using FRAP, we bleached a large region of interest (ROI), shown by the black circle, and quantified the nuclear levels in the center-most bleached nucleus, either in the dark (Fig. 5A,B) or under blue light (Fig. 5D,E). This was repeated with three embryos for each condition, and the recovery was fit to a single exponential (Fig. 5C,F; Koulouras et al., 2018; Cardarelli et al., 2009). The parameter β, which relates to the inverse of the recovery time, was estimated as 0.23 min^−1^, 0.25 min^−1^ and 0.17 min^−1^ for the dark and 0.84 min^−1^, 0.80 min^−1^ and 1.14 min^−1^ for the light. The larger β values in the light indicate that steady state is reached faster in the light and that the flux of DL in and out of the nucleus is greater. Thus, FRAP of DL-mCh-LEXY demonstrates that DL-mCh-LEXY levels recover in the nucleus even when export is favored under blue light, suggesting that DL-mCh-LEXY is rapidly moving in and out of the nucleus.

### Mutation of potential phosphorylation sites in DL C-terminal NES diminishes *sna* but has little effect on *sog*

**Fig. 6.**
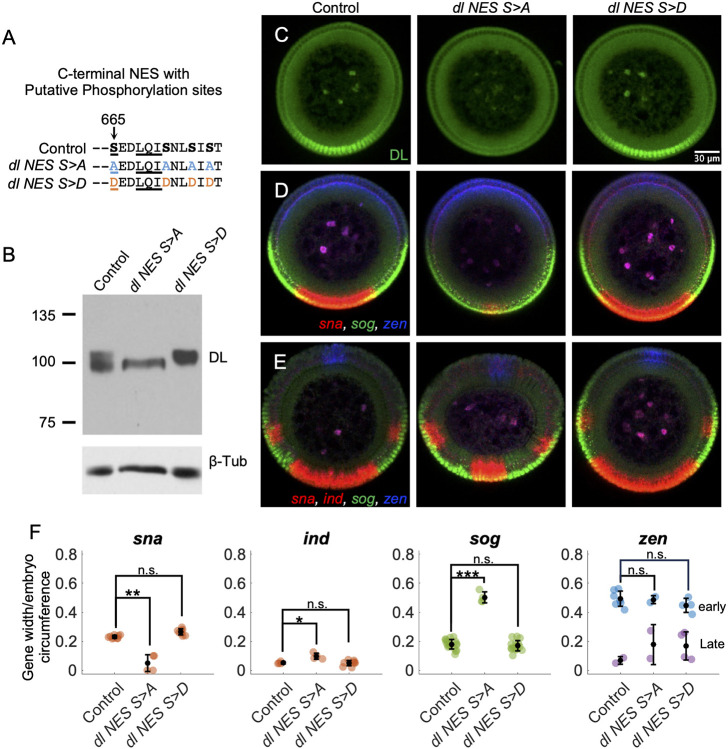
**Mutation of potential phosphorylation sites in the DL C-terminal NES diminishes *sna* but has little effect on *sog*.** (A) Four serine residues (bold) were mutated to either four alanine (blue) or four aspartic acid (orange) residues. S665 has been shown to be phosphorylated (Hilger et al., 2009) and the underlined LQI residues have been shown to be important for the NES to function, as mutation of the L and I residues result in a loss of export (Xylourgidis et al., 2006). (B) Western blot for DL in a *dl* null background each containing *dl-Venus* large rescue constructs: Control (*dl-Venus*), *dl NES S>A* and *dl NES S>D*. (C) Anti-DL staining in Control, *dl NES4 S>A* and *dl NES4 S>D*. (D,E) *In situ* hybridization for *sna* and *ind* (red, same channel; in the Control, *sna* expression is the larger red domain at the bottom of the embryo, and *ind* expression is the two narrower red bands overlapping *sog*), *sog* (green) and *zen* (blue) in Control, *dl NES S>A* and *dl NES S>D* at early nc14 (D) or late nc14 (E). (F) Plot of the quantification of the gene widths normalized by the circumference of the embryo from stainings exemplified by D and E. *sna* and *ind* are in red, *sog* is in green and *zen* is blue at early stages and purple at late stages (mean±s.d.). Tukey's HSD for multiple comparisons after performing one way ANOVA was used, where *P*=9.2×10^−9^ for *sna*, *P*=8×10^−3^ for *ind* and *P*=5.6×10^−18^ for *sog*, when comparing Control to *dl NES S>A*. For the DL antibody staining, the Control had *n*=42, *dl NES S>A* had *n*=31 and *dl NES S>D* had *n*=44. For the *in situ* and quantification, the Control had *n*=7 early and *n*=2 late, *dl NES S>A* had *n*=2 early and *n*=2 late, and *dl NES S>D* had *n*=4 early and *n*=4 late. Note: when *sog* and *ind* exhibited two domains, both domains were included in the average for the quantification of width.

To provide support for the idea that *sog* expression does not change in *dl-LEXY* because export cannot reduce DL levels low enough, we sought alternate ways to increase nuclear export. One way of altering the nuclear export rate is by making mutations in the DL native C-terminal NES (NES4; Xylourgidis et al., 2006). In NES4, a single serine residue (S665) has been identified as a site of phosphorylation through a large mass spectrometry screen (Hilger et al., 2009). As export sequences are largely hydrophobic (Kosugi et al., 2014), phosphorylation of NES4 might act directly to decrease the export rate (McGehee and Stathopoulos, 2024). Other ways phosphorylation could affect the export rate, indirectly, is by masking a NES through regulation of a conformational change or interaction with a binding partner to support nuclear retention. In either case, by blocking phosphorylation, we hypothesized that cytoplasmic DL would increase due to an increase in export (Nardozzi et al., 2010). To be sure all possible sites of phosphorylation in NES4 were removed, we mutated S665 and three other local serine residues. Specifically, the four serine residues were changed to alanine residues (*dl NES S>A*) that cannot be phosphorylated or to aspartic acid residues (*dl NES S>D*) to mimic a constitutively phosphorylated state due to their negative charge (Fig. 6A). These mutations were made using large rescue constructs that include the known regulatory sequences of DL and were assayed at one copy in a *dl^1^/dl^4^* mutant background. Venus was included at the C-terminal end of the *dl* gene in the context of these transgenes to support visualization.

We assayed the *dl NES S>A* and *dl NES S>D* lines using antibody staining and fluorescent *in situ* hybridization. Nuclear DL levels in *dl NES S>A* are clearly decreased compared to the control *dl-Venus* as shown by quantification of DL antibody staining (Fig. 6C; Fig. S5). To confirm that total levels of DL were not affected, we detected DL levels in embryo extracts by western blot using DL antibody (Fig. 6B). We found that the total levels of DL are similar, although the distribution of the migrating band changed in *dl NES S>A*. This loss of differentially migrating bands supports the idea that phosphorylation is disrupted in *dl NES S>A*. The distribution of molecular weights in *dl NES S>D* was observed to shift slightly higher, although it is not as clear as the change in the *dl NES S>A* band.

To determine what effect these changes in the DL gradient had on target genes, we also performed fluorescent *in situ* hybridization against *sna*, *sog*, *zen*, and the laterally expressed gene *intermediate neuroblasts defective* (*ind*), at early nc14 (Fig. 6D) and late nc14 (Fig. 6E) and quantified the results (Fig. 6F). The early and late stages were combined for quantification of the domain width except for *zen*, which changes expression pattern over nc14 (Jaźwińska et al., 1999), and *ind*, which was only detected in late nc14 (Fig. 6D-F; Garcia et al., 2013b). Changes were observed for *sna*, *ind* and *sog* expression in the *dl NES S>A* background (Fig. 6F *sna*, *ind*, *sog*). We did not observe any significant differences in the expression domain widths for the *dl NES S>D* mutant compared to the control. This suggests that the phosphorylated state may be the normal functional state of DL in the ventral region, but DL might still be localized to the cytoplasm by Cactus in more dorsal regions.

Specifically, we observed that the *sna* expression domain is clearly reduced in *dl NES S>A* compared to control (Fig. 6D-F). In addition, *sog* expression expands (Fig. 6D-F *dl NES S>A*), which is expected as Sna represses *sog* and *sna* expression is reduced. *ind* width is slightly larger in the *dl NES S>A* mutant when compared to the control (Fig. 6D-F). These data suggest that affecting the function of a native nuclear export sequence, by mutating putative phosphorylation sites to non-charged residues (Fig. 6A), results in decreased nuclear DL (Fig. 6C and Fig. S5 *dl NES S>A*) that in turn is associated with a reduction in the *sna* domain and expansions of *sog* and *ind* (Fig. 6D-F *dl NES S>A*).

## DISCUSSION

**Fig. 7.**
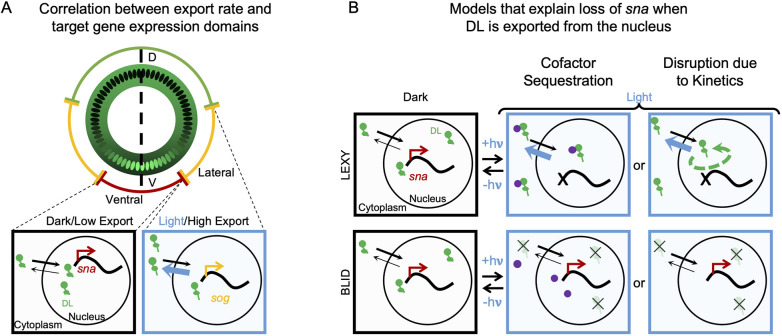
**Models capable of explaining different phenotypes associated with optogenetic perturbation of nuclear DL levels through nuclear export or degradation.** (A) Schematic of expression domains of genes (outer circle: *sna*, red; *sog*, yellow; *zen*, green) around an embryo cross-section along the DV axis also displaying the DL nucleo-cytoplasmic gradient (green). Magnified views of individual nuclei from ventral (left) or lateral (right) regions are shown below and represent a working model of how low levels of export lead to sustained levels of DL sufficient for activation of *sna* (left), while high levels of export lead to low or transient levels of DL only able to activate *sog* (right). *sog* is not expressed stably in ventral regions because Sna represses it. (B) Two possible models to explain differences in *sna* expression detected using DL-LEXY versus DL-BLID. In the dark (left column; ‘−hν) DL-LEXY and DL-BLID (both in green) behave similarly. In one model under blue light, DL-LEXY sequesters a cofactor (purple) in the cytoplasm, while DL-BLID does not affect the cofactor (middle column; ‘+hν). In the second model under blue light, the transient nature of DL-LEXY (green dashed arrow) blocks DL-independent activation of *sna* (red) while degradation of DL-BLID does not (right column; ‘+hν). Thick blue arrows denote increased export rates of DL-LEXY under blue light. Lack of a red arrow marks when transcription is not activated. A thin black ‘x’ indicates DL-BLID molecules that have been degraded by blue light.

The observed differences in target gene responses between *dl-LEXY* and *dl-BLID* provide additional insights into how DL action, specifically DL nuclear level, is interpreted by cis-regulatory systems to pattern the DV axis. We observed that the lowest levels of DL achievable in *dl-LEXY* under blue light are high enough for *sog* expression; whereas with *dl-BLID*, DL is degraded and likely goes below the threshold necessary for *sog* expression under blue light, but only in the very tails of the DL gradient. In contrast, *sna* expression is lost at late nc14 in *dl-LEXY*; while in *dl-BLID*, *sna* expression can remain on (Fig. 2E-J) even though DL levels are higher in *dl-LEXY* than *dl-BLID* (Fig. 4G-J; Movie 5). Lastly, a C-terminal NES serine residue *dl* mutant, resulting in low nuclear DL levels, exhibits decreased *sna* and increased *sog* domain widths but does not affect *zen* domain width. Collectively, these results support the view that a threshold model is insufficient to explain the observed differences in *dl-LEXY*, *dl-BLID* and the S>A mutant and that, in addition to levels, the kinetics of DL import-export play a role in determining the expression of its target genes (Fig. 7A).

Specifically, our results suggest that the kinetics of DL transiting between the nucleus and cytoplasm in *dl-LEXY* disrupts *sna* expression (Fig. 7B ‘Disruption due to Kinetics’). In support of this model, FRAP shows that DL is rapidly transiting in and out of the nucleus in *dl-LEXY* under blue light (Fig. 5). Several other potential explanations were also ruled out. We found no evidence that DL sequesters Twi (Fig. S4). While it is possible that another cofactor is sequestered with DL in *dl-LEXY* under blue light (Fig. 7B ‘Cofactor Sequestration’), this seems unlikely due to a lack of effect on its known interaction partner Twi. One could also imagine that an indirect role could explain these differences. For example, since levels of DL-LEXY are higher under blue light than DL-BLID, a potential neurogenic repressor repressed by Sna that in turn represses *sna* could be active in DL-LEXY but not DL-BLID. In this model, *sna* transcription is lost in DL-LEXY when blue light is applied, and this loss of *sna* results in derepression of a neurogenic repressor, which then represses *sna* and prevents it from reactivating. However, this scenario seems improbable within a 10-20 min timescale. It takes ∼3 min for half the nuclei to stop transcribing *sna* transcripts, which have a half-life of 13.6 min and estimated copy number of ∼180 copies/cell (Boettiger and Levine, 2013). Although Sna protein half-life is unknown, based on estimated rates of translational elongation (Chen et al., 2024; Dufourt et al., 2021) and initiation (Dufourt et al., 2021), it can be produced in about 24.1-110.5 s given its length (390 aa). Therefore, Sna protein would likely still be present in this timeframe. Additionally, transcriptional bursts yield an average of tens of transcripts per minute (Bothma et al., 2014; Fukaya et al., 2016), meaning it would take 10+ minutes to make 100 or more transcripts of this invoked neurogenic repressor. Thus, the timeline for removing Sna and activating a neurogenic repressor seems unlikely within 10-20 min. For all these reasons, we favor the interpretation that DL nuclear import-export kinetics can influence target gene expression.

While our study focused on two threshold responses relating to *sna* and *sog* target genes, there is still more to learn with regard to other target genes. For example, we found that *zen* does not respond to DL levels in either *dl-BLID* or *dl-LEXY* when illuminated with blue light from nc13 to nc14 (Fig. S3). This is surprising because *sog* and *zen* are thought to share the same threshold. One potential explanation is that *zen* responds to DL at a timepoint before we imaged (e.g. nc12). Alternatively, DL-repressor activity may be fundamentally different from DL-activator activity despite similar concentration-thresholds. For example, if DL binds more tightly to sites where it acts as a repressor, DL-LEXY and DL-BLID might be unable to support export or degradation at such sites, especially if degradation is primarily happening in the cytoplasm. In future studies, it would be of interest to focus on how export and degradation affect DL repressor activity, which may require earlier perturbations or have no effect at all.

This study sheds light on the timing of morphogen action and highlights the importance of nuclear import-export kinetics, supporting recent models (Barros et al., 2021; Schloop et al., 2020). These findings suggest a broader principle regarding how gene expression responds to morphogen dynamics, emphasizing that it is influenced not only by concentration but also by the temporal presentation of morphogens. For example, transient levels required for low-threshold targets may actively prevent the transcription of high-threshold targets, ensuring the correct positioning of expression domains. Current interpretations often assume a uniform concentration of TFs throughout the nucleus. Therefore, future super-resolution imaging of DL could reveal how variations in local DL concentrations, influenced by import/export dynamics, affect gene expression.

Additionally, future research should explore TF binding site affinity and cooperative binding using optogenetics (Kim et al., 2024; Rao et al., 2021). Previous studies in yeast have examined how TF dynamics encode and decode information, with one optogenetic study indicating that certain targets are regulated by high thresholds (low affinity sites) for greater robustness (Sweeney and McClean, 2023). Combinatorial control may also make genes less sensitive to concentration changes; for example, *sna* lacks many linked DL and Twi sites, unlike DL target genes in lateral regions (Markstein et al., 2004; Reeves and Stathopoulos, 2009). Furthermore, studies have focused on whether stochastic gene expression is instructive or buffered against variability (Exelby et al., 2021; Naqvi et al., 2023; Boettiger, 2013). *sog* expression appears to be stochastic (Reeves et al., 2012; MacNamara, 2014), while *sna* exhibits coordinate expression (Lagha et al., 2013; Boettiger and Levine, 2013). Optogenetic tools could further elucidate the possibly differing temporal requirements for inputs that support stochastic versus coordinate gene expression.

In addition to categorizing gene expression responses supported by morphogens in *Drosophila* and other higher animals by their thresholds (high, medium, low), these responses should also be classified by their dynamical requirements, such as sustained, transient or absent/reduced morphogen input. Cis-regulatory systems may be tuned to detect sustained levels for high-threshold targets, transient levels for low-threshold targets, and reduced levels for targets repressed by the morphogen (reviewed by Irizarry and Stathopoulos, 2021). Moreover, enhancers with varying dynamic requirements have been shown to coordinate gene expression during *Tribolium* development (Mau et al., 2023). Future studies could also explore whether dynamics are sensed by enhancers and/or promoters (Hoppe et al., 2020; Delás et al., 2023). In the future, we anticipate the field will discover more dynamic regulatory paradigms through the use of optogenetic tools, providing further insights into how morphogens work.

While optogenetic tools offer precise control of transcription factor levels, they have limitations. Both DL-LEXY and DL-BLID produce different peak levels and widths of the gradient, with significant differences noted for DL-BLID when assayed by antibody staining. This variability is likely due to their different sensitivities to light, as tags may be leaky even in the dark and because embryos are inadvertently exposed to some ambient light during fixation. Despite these limitations, both tags are able to support high threshold target gene expression. Thus, the effects of light perturbations always have to be interpreted in the context of the dark condition, which – to be clear – is not exactly wildtype. Another limitation is that transcription and DL levels are measured in different embryos/lines and not within the same nucleus. Future experiments using far red fluorophores, white light lasers or two-photon microscopy could help address these limitations related to the activation wavelengths of the optogenetic tags (∼400-500 nm).

## MATERIALS AND METHODS

### Fly stocks and husbandry

All *D. melanogaster* stocks were kept at 22°C in standard medium. Experimental crosses were kept in cages with apple juice agar plates supplemented with yeast paste and were kept at 18°C. Embryos were collected for less than 2 weeks, such that parents were 1-14 days old at time of collection. *w; dl-LEXY/CyO; PrDr/TM3* and *w; dl-BLID/CyO; PrDr/TM3* were crossed to *Sp/Cyo; MCP-mCherry (w+, NLS)/TM3 (**Bothma* et al., *2018**)* to generate *w; dl-LEXY/CyO; MCP-mCherry (w+, NLS)/TM3* and *w; dl-BLID/CyO; MCP-mCherry (w+, NLS)/TM3*, which were grown in bottles, and virgin *dl-LEXY; MCP-mCherry (w+, NLS)/TM3* or *dl-BLID; MCP-mCherry (w+, NLS)/TM3* were selected. These virgins were crossed to males bearing the MS2. MS2 lines included *sna-MS2 BAC (III)*, *sog-MS2 (I); Sp/Cyo*, and *Sp/Cyo; zen-MS2/TM3 (III)*. *sna-MS2* is a large reporter construct of ∼25 kB with MS2 inserted at the 5′ end of the transcript following the 5′ untranslated region (UTR) and the coding sequence replaced by the gene *yellow* (Perry et al., 2010; Bothma et al., 2015). Plasmid DNA from (Bothma et al., 2015) was inserted on the third chromosome at 65B2; 3L:6442676 (Irizarry et al., 2020). *sog-MS2* and *zen-MS2* contain insertions of MS2 within introns. In addition, *dl-mCherry-LEXY/CyO* and *dl-mCherry-BLID/CyO* were grown in bottles and homozygous mothers and fathers were added to experimental cages*. y^2^ cho^2^ v^1^ P{nos-phiC31\int.NLS}X; attP2 (III)* (NIG-FLY TBX-0003) was used to make *y^2^ cho^2^ v^1^ P{nos-phiC31\int.NLS}X; P{dl-gRNA}attP2 (III). y^2^ cho^2^ v^1^; Sp/CyO, P{nos-Cas9, y+, v+}2A* (NIG-FLY CAS-0004) virgins were crossed to *y^2^ cho^2^ v^1^ P{nos-phiC31\int.NLS}X; P{dl-gRNA}attP2 (III)* for injection. See Table S1 for a complete list of lines used. The CRISPR/Cas9 lines generated in this study (*dl-LEXY*, *dl-mCh-LEXY*, *sog-MS2* and *zen-MS2*) contain a DsRed marker.

### Homologous repair template cloning

*LEXY* (Bonger et al., 2014) was codon optimized and, along with *MS2* (Yamada et al., 2019), synthesized by GenScript in pUC57. The *dl-LEXY*, *dl-mCh-LEXY*, *sog-MS2* and *zen-MS2* homologous repair templates were generated by editing *pHD-DsRed* (Gratz et al., 2014). The right homology arm for *dl-LEXY and dl-mCh-LEXY* was generated by PCR using a *dl-Venus-BAC* (Reeves et al., 2012) as a template, and was inserted into *pHD-DsRed* downstream of the *DsRed* using BglII and XhoI sites. The left homology arm was generated by overlap PCR, combining three fragments: the C-term of *dl*, the LEXY domain, and the *dl* 3′ UTR. The left homology arm of *dl-mCh-LEXY* was made by overlap PCR, combining PCR products that used *dl-mCherry* HDR and the *dl-LEXY* HDR as a template. This PCR product was inserted into *pHD-DsRed* upstream of the *DsRed* using EcoRI and NheI sites. The *sog-MS2* homologous repair template was made by PCR, using a BAC as the template (BacPac Resource Center, BACR25D05). Overlap PCR was used to mutate the gRNA binding site in the repair template. The left homology PCR product was cut with NheI and AseI and the *pHD-DsRed* plasmid was cut with NheI and NdeI to make compatible sticky ends, which were ligated together. The right homology arm PCR product and the pHD-DsRed were digested with AscI and XhoI and ligated. The *zen-MS2* homologous repair template was made the same way as the *sog-MS2* template but used NheI and NdeI on both the insert and the backbone, and the right homology arm also used overlap PCR to mutate the gRNA sequence. The *MS2* sequence was added using NotI and AvrII, which were added to the reverse primer used to generate the left homology arm of both *sog-MS2* and *zen-MS2*.

The *zen-MS2* gRNA was made by BbsI digestion of pCFD5 and Gibson assembly was used to combine the vectorized backbone and the PCR product. In both *sog-MS2* and *zen-MS2*, the MS2 sequence was inserted into the first intron, as annotated on Flybase. See Table S1 for a complete list of primers used.

### CRISPR/Cas9 genome editing

For *dl-LEXY* and *dl-mCh-LEXY*, *y^2^ cho^2^ v^1^; Sp/CyO, P{nos-Cas9, y+, v+}2A* virgins were crossed to *y^2^ cho^2^ v^1^ P{nos-phiC31\int.NLS}X; P{dl-gRNA}attP2 (III)*. The HDR template for *dl-LEXY* and *dl-mCh-LEXY* were injected into embryos from this cross. The *sog-MS2* HDR was co-injected with a previously made gRNA (Dunipace et al., 2019) into w[1118]; PBac{y[+mDint2]=vas-Cas9}VK00027 (Bloomington *Drosophila* Stock Center, #51324). For *zen-MS2*, gRNAs were found using flyCRISPR Target Finder (Gratz et al., 2014). The *zen-MS2* HDR was co-injected into *y2 cho2 v1; attP40{nos-Cas9}/CyO (NIG-FLY CAS-0001)*. For both *sog-MS2* and *zen-MS2*, Rainbow Transgenics performed the injections. All HDR templates included DsRed as a selectable marker, and transgenics were screened for DsRed expression.

### Live imaging

Embryos from crosses between *dl-LEXY* females and males from the *MS2* line were collected for 4 h or overnight, both at 18°C. To prepare the embryos for live imaging, embryos were hand dechorionated in the dark, using a red film (Neewer, 10087407). Embryos were transferred to an agar square and oriented so that the face that would be imaged was facing the agar. Preprepared slides were made by adding heptane glue (heptane plus double sided tape) to a coverslip that was taped to the slide and allowing it to sit overnight. This slide was used to pick the embryos up from the agar. Embryos were then checked to make sure the orientation had not been disrupted and oriented again if necessary. Water was then added to prevent desiccation of the embryos. Embryos were transferred to the microscope in a covered box. Imaging occurred on a Zeiss LMS 800 using a 25× immersible objective (LCI Plan-Neofluar 25×/0.8 Imm Korr DIC M27) at 1.7 zoom. The MCP-mCherry signal was detected using a 561 nm laser at 1% laser power with 800 V gain on a GaAsP PMT detector. The 488 nm laser at 4.5% laser power was used to perform blue light illumination with 500 V gain on a GaAsP PMT detector to protect the detector from the high laser power. *Z*-stacks were taken, with 30 *z*-planes per time point at 1 µm thickness. Images were taken every ∼25 s, starting as soon as the previous *z*-stack finished. Images were captured as 16 bit images, and each *z*-slice was 512×512 pixels, with each pixel being 0.29 µm in length and width. DL-mCh-LEXY and DL-mCh-BLID were imaged using the same settings as the MS2/MCP imaging, except the laser power of the 561 nm laser was 2% instead of 1%. Upon beginning imaging, embryos were staged under red light (white light covered with a red filter). Embryos that were the right stage and orientation were imaged. Orientation was determined by the signal being imaged. For DL-mCh-LEXY, DL-mCh-BLID and *sna-MS2*, this meant that the signal domain was centered. For *sog-MS2* and *zen-MS2* this meant detecting the boundary of the expression domain. FIJI/ImageJ was used to visualize images and to save movie files (Schindelin et al., 2012).

### Fluorescence recovery after photobleaching

FRAP was imaged similarly to previous imaging setups. Images were 512×512 pixels, 16 bit, and taken on a Zeiss LMS 800 using a 25× immersible objective (LCI Plan-Neofluar 25×/0.8 Imm Korr DIC M27) at 5.0 zoom with a pixel size of 0.100 µm. The DL-mCh-LEXY signal was detected using a 561 nm laser at 1% laser power with 900 V gain on a GaAsP PMT detector. The 488 nm laser at 4.5% laser power was used to perform blue light illumination with 500 V gain on a GaAsP PMT detector to protect the detector from the high laser power. Time points were acquired at a rate of one frame per 2 s. Embryos were illuminated with blue light starting at time point six (∼18 s) and remained on until the end of imaging at ∼20 min. Bleaching was performed starting at time point 21 and ending at time point 40 (∼1 min) in an ROI using the 561 nm laser at 20% laser power and performing 50 iterations of bleaching at each time point.

### Fixed imaging

For fixed sample preparation of the *dl-Venus*, *dl NES S>A* and *dl NES S>D*, embryos were collected for 1 h, aged 2-3 h and then were dechorionated in bleach, fixed in 4 ml of 9.25% formaldehyde and 4 ml of heptane for 20 min and then rinsed and stored in methanol at −20°C. For *in situ* hybridization, protocols were followed as described previously (Kosman et al., 2004) using riboprobes generated for *sna*, *ind*, *sog* and *zen*. Sheep anti-digoxigenin (Life Technology, PA185378), rabbit anti-FITC (Invitrogen, A889) and mouse anti-Biotin (Invitrogen, 03-3700) were used (1:400). Fluorescently conjugated secondary antibodies, Alexa 555, 488 and 647, from Thermo Fisher Scientific were used (1:400). See Table S1 for a complete list of reagents used. Mouse anti-dorsal 7A4 [Developmental Studies Hybridoma Bank (DSHB); Whalen and Steward, 1993] was used for DL antibody staining (1:10), following the same protocol as FISH. Embryos that were the right stage based on visualization of a nuclear stain (DAPI) were imaged and staging was confirmed by *zen* and *ind* expression. Fixed sample preparation for DL-LEXY and DL-BLID were performed the same way. During processing, embryos were shielded from ambient light as much as possible; however, embryos were exposed to light, which may have affected DL levels in these embryos. This effect would be greater in DL-BLID, which cannot recover, unlike DL-LEXY, which is reversible.

### Western blot

For western blot analysis, embryos were collected and staged under white light. Embryos at mid nc14 were then added to a tube containing SDS buffer, ruptured using a fine needle and homogenized. Embryo extracts were then run on a discontinuous SDS-PAGE gel and transferred to 0.45 µm Immobilon-P PVDF. Chemiluminescent detection was performed using a DL antibody (anti-Dorsal 7A4, DSHB, final concentration of 480 ng/ml in 4 ml total volume; Whalen and Steward, 1993), and β-tubulin (E7, DSHB, final concentration of 590 ng/ml in 4 ml total volume; Chu and Klymkowsky, 1989) antibody was used as a loading control.

### Quantification and statistical analysis

#### Sites of transcription detection and quantification

To quantify the number of MS2 foci, or sites of transcription, in the images/movies captured, three custom MATLAB functions (https://github.com/StathopoulosLab/McGehee_2024) were used. The first function opens the image, including relevant metadata, and performs the foci detection. First ‘salt and pepper’ noise is removed using a median filter. The background is subtracted by using a median filter over a larger area to blur the image and then subtracting the blurred image from the original. After background subtraction, the image is blurred with a Gaussian filter. The image is then thresholded by a user defined threshold, tiny objects of only one pixel are removed, and objects detected on the edge are removed. The entire embryo is segmented by projecting all the time points together, blurring the image with a Gaussian filter and using thresholding. The detected embryo is then morphologically closed to smooth the edges and small objects less than 100 pixels are discarded so only one object, the embryo, is detected. Any focus detected outside the embryo is removed. We observed that the background intensity of nuclei increased over time and, to account for this, we increased the threshold by a small amount using a user defined value that increases logarithmically during nc14. To increase the detection of foci, we segmented the unprocessed image a second time using a user defined threshold and retained only the foci detected with both thresholds. The algorithm works by setting the first threshold low, detecting both real foci and noise, and then removing the noise based on a second, higher threshold. The centroid coordinates for these foci are saved for further analysis.

A second function displays the images in a graphical user interface and allows overlapping the mask of the segmented foci on the image. In addition to overlaying, the centroids can be used to plot points at the detected foci. These were used to evaluate the success of the thresholding. Comparable imaging conditions used the same empirically determined threshold. Specifically, when comparing dark and light or *dl-LEXY* and *dl-BLID* the same threshold was used. The threshold was only changed for different MS2 signals (i.e. *sna* versus *sog*) or different lengths of imaging (i.e. nc12-nc14 versus 25 min of nc14). A third function was used to quantify the number of foci and plot the results. To plot the averages, the data was interpolated using the MATLAB built in interp1 function and the Modified Akima cubic Hermite interpolation method. The mean number of foci and standard error of the mean were calculated from the interpolated data and plotted. In addition, this function also approximated the area of expression. This was done by concatenating all the centroids in given time windows, corresponding to nc13 or the first 100 time points of nc14, and removing foci that were two median absolute deviations (MAD) from the median for the centroids of all foci detected. We used a conservative approach because this removed points that tended to be isolated, were not detected in multiple frames, or were actually noise and not a real focus. To determine the area, a convex hull was drawn around the remaining points and the area for this convex hull was determined. The area at nc13 was then subtracted from the area at nc14 to determine the change in nc13 to nc14 and account for potential discrepancies in the orientation of the embryo. This was only done for *sog-MS2* and *zen-MS2*.

To determine whether the differences in area were statistically significant, we performed one way ANOVA and Tukey's honestly significant difference (HSD) test for multiple comparisons to compare *dl-LEXY* dark, *dl-LEXY* light, *dl-BLID* dark and *dl-BLID* light. We performed this analysis for the areas determined for both *sog-MS2* and *zen-MS2*. A *P*-value less than 0.05 was considered statistically significant.

#### Live fluorescent protein quantification

To quantify DL-mCh-LEXY, DL-mCh-BLID (Fig. 4) and Twi-LlamaTag-mCh (Fig. S4) nuclear levels, images were segmented using the MATLAB edge function and a Laplacian of Gaussian with a standard deviation of four for the filter. Water-shedding was performed using the MATLAB watershed function to disconnect any nuclei that were connected. Objects were filtered by size to remove large (i.e. two connected nuclei) or small (i.e. non nuclei or partial nuclei) objects. During time points when no nuclei were detected (such as when blue light was turned on or off), the image segmentation from the previous time point was used. The embryo was detected by blurring the image and using a normalized threshold of 0.005, followed by morphological opening and closing. The boundaries of the embryo were detected and used to fit an ellipse. From the ellipse, the midline was determined and only nuclei within 100 pixels above or below the midline were included for analysis. The average intensity was calculated for each individual nucleus at a given time point and then was averaged together for each time point and plotted as the mean±s.d. To quantify individual nuclei (Fig. 1) a similar procedure was used, except the standard deviation for the Laplacian of Gaussian was ten and the embryo and midline were not detected due to the high level of zoom. In addition, individual nuclei were tracked by computing the distance between nuclei centers between time points. The minimum distance under a threshold (set as the radius of a single nucleus) was used to join tracks. In the event a nearest neighbor was not found, previous time points were used to find similar positioned nuclei up to ten time points away. Tracks that were missing the first ten time points, last ten time points, or had less than half the total number of time points were removed. This ensured that enough data was provided to the fitting algorithm. Fitting was performed as described below for FRAP.

#### FRAP quantification

To quantify DL-mCh-LEXY levels during FRAP, the center most nucleus in the bleach ROI was tracked over time. Image segmentation was performed using the MATLAB edge function and a Laplacian of Gaussian with a standard deviation of ten for the filter. Water-shedding was performed using the MATLAB watershed function to disconnect any nuclei that were connected. In the event that the nucleus was unable to be segmented, the segmentation of the previous time point was used. Tracking was performed by calculating the distance between objects at two different time points and using the smallest distance to join tracks. The recovery after bleaching was fit to the equation

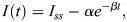
where *I(t)* is the fluorescence intensity at time *t*, *I_ss_* is the steady state intensity, α is the difference between the initial intensity and the steady state intensity, and β is the inverse recovery time. β is likely related to the import and export rates, which can be shown explicitly for simple models of import-export kinetics (Cardarelli et al., 2009; Williamson et al., 2021; Koulouras et al., 2018). Larger β is indicative of faster cycling in and out of the nucleus such that steady state is reached sooner. Fitting was performed only on the recovery period of the curve, after bleaching. A two-sample unpaired *t*-test was performed to determine whether the mean β was significantly different between FRAP performed on embryos kept in the dark or illuminated with blue light.

#### Fixed DL gradient quantification

To quantify the DL gradient in fixed samples, images were inputted into a previous developed function which measures the intensity of DL along the DV axis and fits the DL gradient to a Gaussian function (Trisnadi et al., 2013).

#### Fixed gene expression domain width quantification

To quantify the gene expression patterns, first the ring of nuclei was segmented by using the MATLAB edge function and a Laplacian of Gaussian with a standard deviation of 20 for the filter on the DAPI or histone channel. The two largest filled objects were taken, which are the outer and inner ring of the nuclei. Ellipses were fit to both of these rings, and the average ellipse parameters were taken to get the ellipse centered between these two rings. The channels for the gene expression were segmented using a threshold and the largest objects were used to remove background. The domain width was determined as the points where the ellipse crossed the segmented objects of gene expression. To calculate arc length, the equation of an ellipse was integrated between these two points. The perimeter was calculated by integrating around the entire ellipse. The domain width was then normalized by the perimeter. The widths were averaged together across embryos, and if a pattern contained two domains, both widths were included in the average. Tukey's HSD for multiple comparisons after performing one way ANOVA was used to determine whether the means were significantly different between experimental and control conditions.

#### Power analysis

To determine the sample size, a power analysis was done for each experiment. For the number of MS2 transcription sites in *sna-MS2* a power analysis using a predicted mean of 200 transcription sites, standard deviation of 40, power of 0.80 and significance value of 0.05 for a two-sample unpaired *t*-test was carried out in MATLAB using sampsizepwr. This resulted in a sample size of *n*=3. For the change in area of *sog-MS2* and *zen-MS2*, this procedure was repeated using a mean area of 2000, standard deviation of 1500, power of 0.80 and significance value of 0.05 for a two-sample unpaired *t*-test. This also resulted in a sample size of *n*=3. Similarly, we performed this analysis for the quantification of the DL gradient using a mean amplitude of 100, standard deviation of 25, power of 0.80 and significance value of 0.05 for a two-sample unpaired *t*-test. This resulted in a sample size of *n*=6.

## Supplementary Material



10.1242/develop.202775_sup1 Supplementary information

Table S1. A list of the *Drosophila melanogaster* lines, primers, plasmids, reagents, and software used in this study.
