# Supplementary material for: Target gene responses differ when transcription factor levels are acutely decreased by nuclear export versus degradation
Source: Development. 2024 Nov 8;151(21):dev202775. doi: 10.1242/dev.202775 (PMC11574349; doi:10.1242/dev.202775)
Supplement: Supplementary information [file develop-151-202775-s1.pdf]

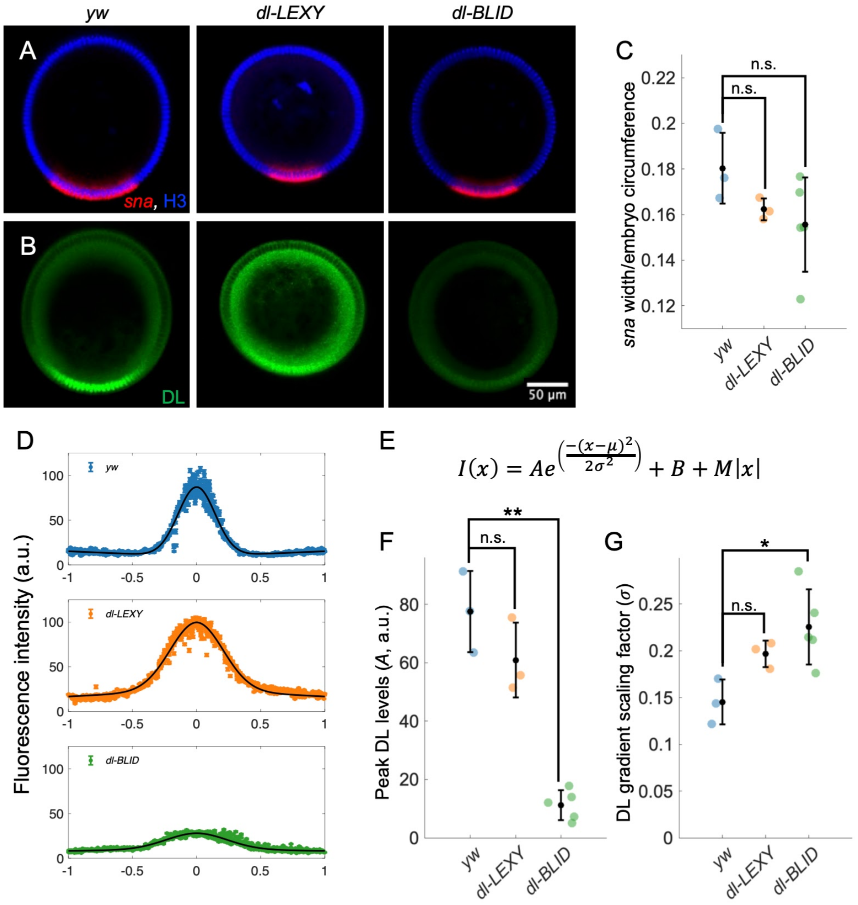

Fig. S1. DL peak levels are lower in *dl-BLID* but not *dl-LEXY,* and *sna* expression width is not different in the dark. (A) *sna* expression (red) by in situ and Histone (H3) antibody staining (blue) in *yw*, *dl-LEXY*, and *dl-BLID* in the dark. **(B)** DL antibody staining (green) in *yw*, *dl-LEXY*, and *dl-BLID* in the dark. **(C)** Quantification of the *sna* domain width in A for *yw*, *dl-LEXY*, and *dl-BLID*. Individual embryos are shown. *yw* is in blue, *dl-LEXY* in orange, *dl-BLID* in green, and mean ± s.d. in black. Although the average width of *sna* is less in *dl-LEXY* and *dl-BLID*, these differences are small and not statistically significant (p = 0.43 for *dl-LEXY* and p = 0.17 for *dl-BLID*, when compared to *yw* using Tukey's HSD for multiple comparisons after performing one way ANOVA). **(D)** The plots of the quantification of DL levels from B. The colored markers denote DL levels in nuclei, and the black lines are the

Development • Supplementary information

best fit to a Gaussian function. **(E)** The Gaussian function that the DL gradient quantification is fit to. **(F)** The peak levels of DL in *yw*, *dl-LEXY*, and *dl-BLID* (p = 0.17 for *dl-LEXY* and p = 4.96 * 10^{-5} for *dl-BLID*, when compared to *yw* using Tukey's HSD for multiple comparisons after performing one way ANOVA). **(G)** The scaling factor (σ) for the DL gradient in *yw*, *dl-LEXY*, and *dl-BLID* (p = 0.18 for *dl-LEXY* and p = 0.02 for *dl-BLID*, when compared to *yw* using Tukey's HSD for multiple comparisons after performing one way ANOVA). The scaling factor (σ) is a measurement of gradient width, and the the tails in D look slightly wider. Fixed embryos were shielded from light as much as possible, but they were exposed to ambient light during processing and fixation. Since DL-LEXY is reversible and DL-BLID is not, this may have a greater effect on DL-BLID and could partially account for why the levels are so low in DL-BLID compared to the control.

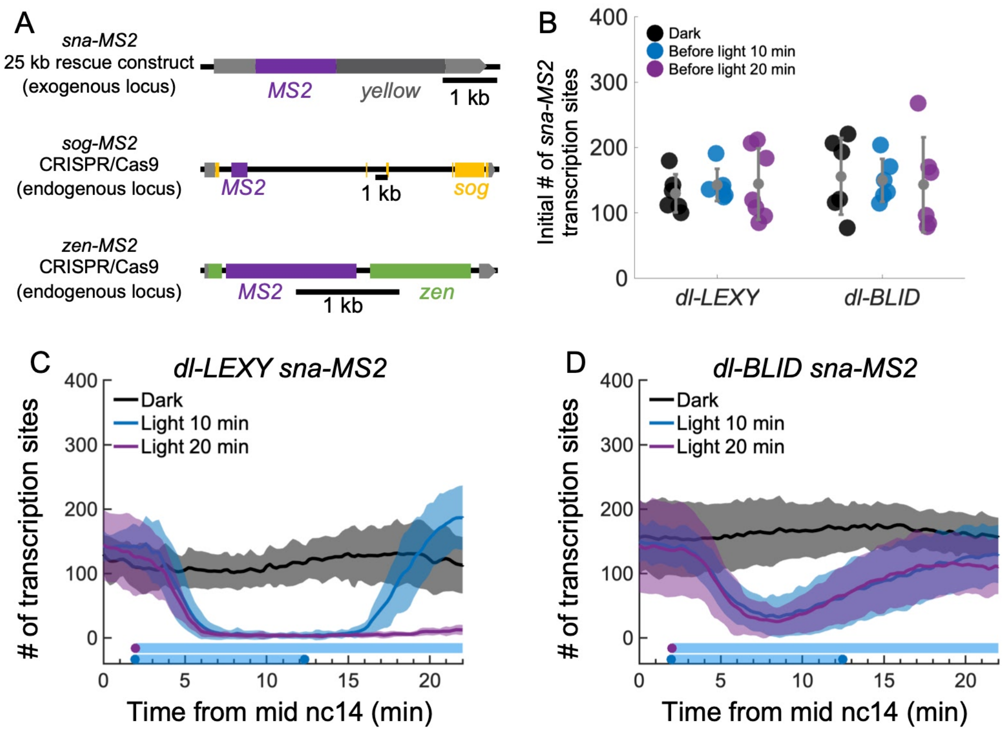

Fig. S2. MS2 reporter constructs and variability in starting number of transcription sites for *sna-MS2*. (A) The MS2 constructs used for observing active transcription live. The *sna-MS2* line was from a previously published study (Bothma et al. 2015) whereas the *sog-MS2* and *zen-MS2* reporters were created in this study. The *sna-MS2* reporter is a large reporter construct (~25 kB) inserted as an exogenous copy on the 3rd chromosome. Shown here is just the MS2 and exons of the *sna-MS2* reporter. The *sog-MS2* and *zen-MS2* are lines with MS2 inserted at the endogenous loci using Crispr/Cas9 (see Methods). **(B)** The starting number of transcription sites for *dl-LEXY* and *dl-BLID* with markers color coded to match each condition: dark (black), before light 10 min (blue), and before light 20 min (purple). The number of transcription sites did not have a statistically significant difference between the means (p = 0.96 for one way ANOVA and individual comparisons were not significant by Tukey's HSD for multiple comparisons, in black mean ± s.d., n = 6 for all conditions except *dl-LEXY* light 20 min

Development • Supplementary information

where n = 7). **(C,D)** The unnormalized mean number of transcription sites for *dl-LEXY* (C) and *dl-BLID* (D) in the dark (black), with 10 min of blue light (blue), or 20 min of blue light (purple). The number of initial sites of *sna* active transcription can vary for multiple reasons. One reason is that both BLID and LEXY are leaky in the dark, and this could lead to differences in DL levels which affect the number of active sites. These embryos are staged by tracking the cellularization front and imaging begins when the front is 50% the length of nuclei (mid to late nc14). This staging technique could lead to variability in the starting number of transcription sites for *sna*, as *sna* sites of active transcription are known to vary over time (Bothma et al. 2015). Embryos were collected from the same respective cages either on the same day or subsequent days.

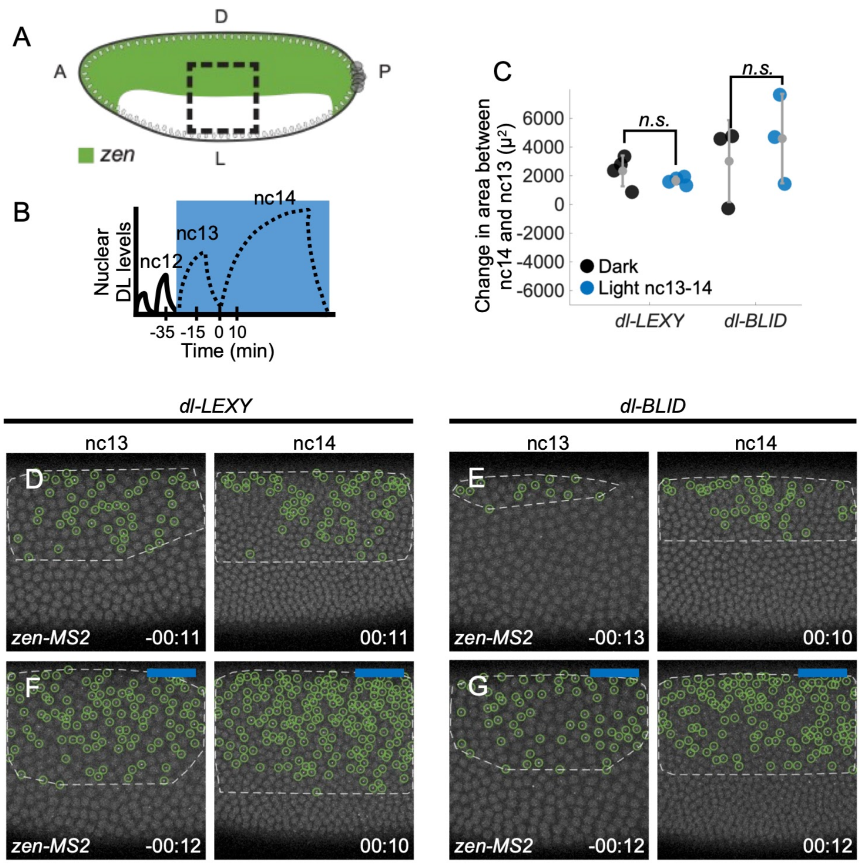

Fig. S3. In both *dl-BLID* and *dl-LEXY*, no change in the *zen-MS2* boundary is observed when comparing nc13 to nc14. (A) A schematic of *zen* expression with the field of view and blue light illumination window marked by the dashed black box. **(B)** The blue light illumination window from nc13 through the end of nc14. **(C)** Quantification of the change in area between nc14 and 13 for *zen-MS2* in *dl-LEXY* and *dl-BLID*. Black markers represent the dark and blue markers represent illumination from nc13-14 (in gray, mean ± s.d., n = 3 for *dl-BLID* and n = 4 for *dl-LEXY*). When comparing dark to light at nc13-14, the means are not significantly different, p = 0.96 for *dl-LEXY* light vs. dark and p = 0.77 for *dl-BLID* light vs. dark (Tukey's HSD for multiple comparisons after performing one way ANOVA). **(D-G)** *zen-MS2* in *dl-LEXY* (D,F) and *dl-BLID* (E,G), when kept in the dark (D,F) or when illuminated continuously from

Development • Supplementary information

nc13 through nc14 (F,G) at nc13 (-00:11, -00:12, and -00:13) and early-nc14 (00:10, 00:11, and 00:12). Variability in the change in area between nc14 and 13 is higher in *dl-BLID* and it appears that between nc13 and nc14 the *zen* domain is expanding. However, this expansion and the variability result in no statistically significant difference between the light and the dark. This may have to do with how *zen* senses DL. In *dl-LEXY,* the low levels of DL are likely sufficient for repressing *zen*, but in *dl-BLID*, the leaky nature of *dl-BLID* might result in different levels of DL and *zen* might be sensitive to this. Dorsal-lateral views are shown. Embryos of a certain genotype were collected from the same cage either on the same day or subsequent days.

Development • Supplementary information

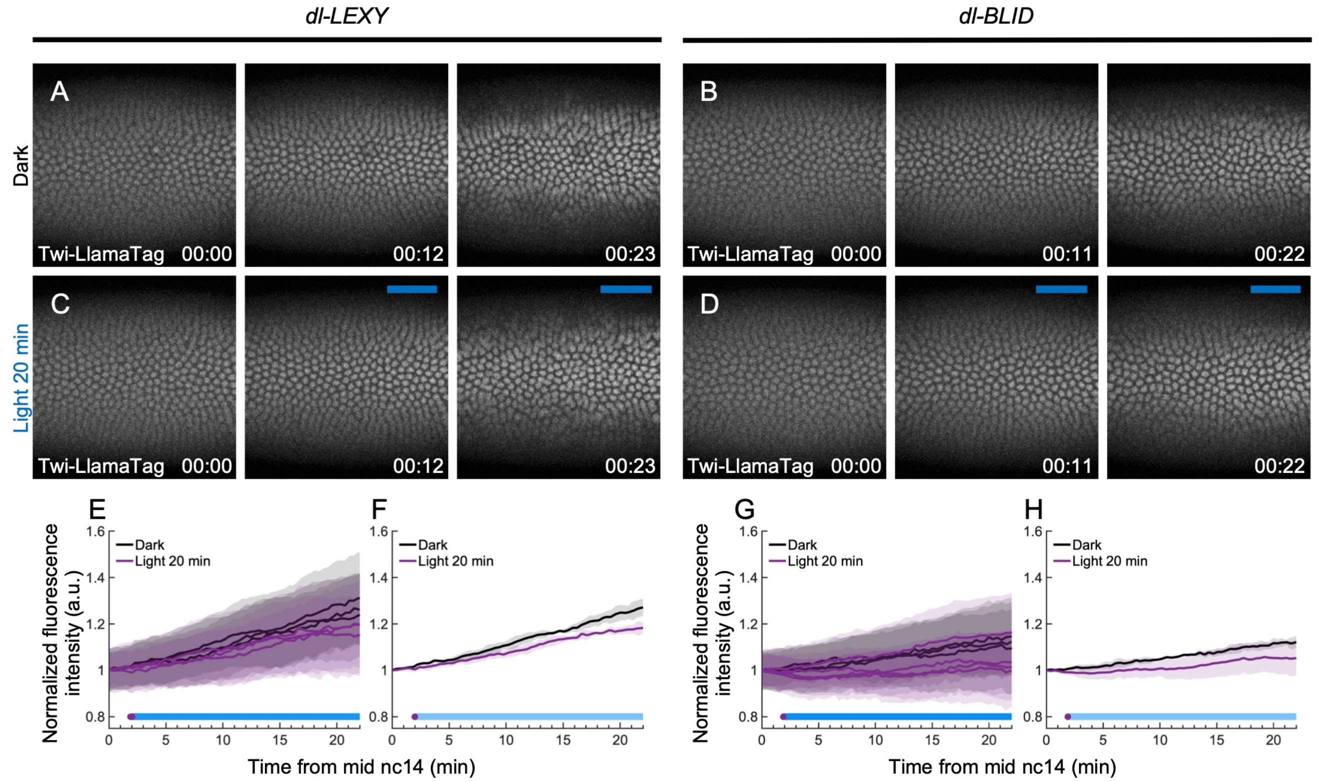

Fig. S4. Twi levels do not change appreciably at late nc14 under blue light in *dl-LEXY* or *dl-BLID*. See also Movie 8. (A, B) Twi-Llama-Tag bound to mCherry in *dl-LEXY* (A) and *dl-BLID* (B) in the dark. **(C,D)** Twi-Llama-Tag bound to mCherry in *dl-LEXY* (C 00:12 and 00:23) and *dl-BLID* (D 00:11 and 00:22) after blue light exposure. **(E,F)** Plots of the mean fluorescence of Twi-Llama-mCherry for individual embryos normalized by the starting intensity (mean ± s.d. across nuclei, n = 3 for both). The black lines are individual embryos kept in the dark and the purple lines are individual embryos with 20 min of blue lighti. **(G,H)** Plots of the mean fluorescence of Twi-Llama-mCherry when averaging embryos together (mean ± s.d across embryos., n = 3 for both). In both *dl-LEXY* and *dl-BLID*, the effect of blue light on the levels of Twi is minimal, while there is a slight decrease in the mean intensity when averaging embryos together, this falls within the standard deviation when averaging individual nuclei per embryo.

Development • Supplementary information

Thus, a change in Twi levels does not explain the difference in *sna* expression between *dl-LEXY* and *dl-BLID* under blue light. In addition, since little to no effect on nuclear levels of Twi was observed when embryos were illuminated with light and Twi levels did not increase in the cytoplasm, this result suggests that export of DL from the nucleus is unlikely to cause the export of other transcriptional cofactors to inhibit *sna* expression indirectly. Ventral views are shown. Embryos were collected from the same cage either on the same day or subsequent days.

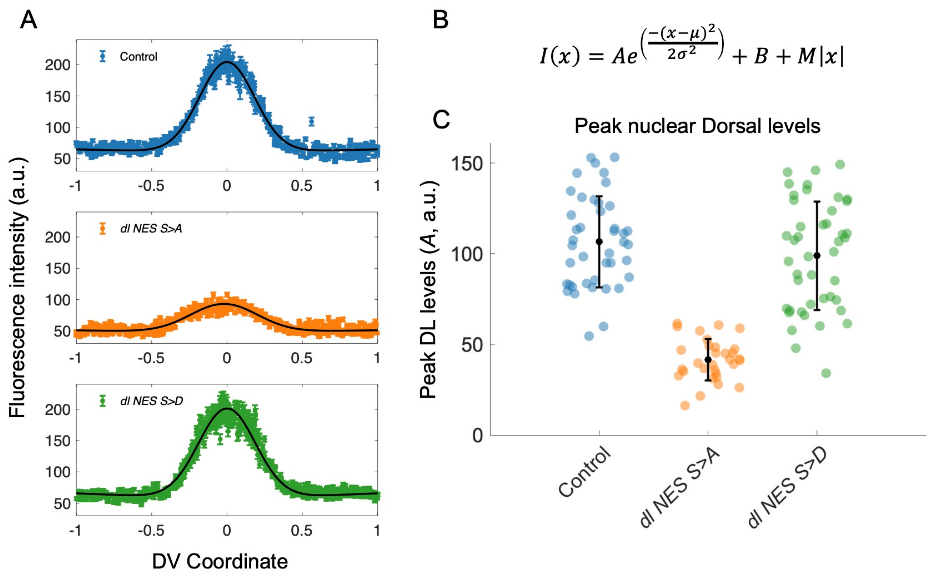

Fig. S5. Peak levels of DL are lower in *dl NES S>A* than in Control embryos. (A) Fluorescent intensity levels in antibody stainings for DL are calculated for each individual nucleus (colored markers). Each plot is a single, representative embryo from the Control (*dl-Venus; blue*), *dl NES S>A (orange)*, and *dl NES S>D (green)*. These traces are aligned so that peak levels of DL correspond to the 0 DV coordinate. The fluorescent intensity is then fit to the equation in B (black lines). **(B)** The Gaussian function that the DL gradient is fit to. *A* represents the amplitude, or the highest levels of DL. **(C)** A plot of the amplitudes, *A*, or peak levels of DL for the Control (*dl-Venus; blue*), *dl NES S>A (orange)*, and *dl NES S>D (green)*. In black is the mean ± s.d. The levels in *dl NES S>A* are lower than the Control or *dl NES* S>D (p = 8.6 * 10^{-20} and 7.8 * 10^{-17}, Tukey's HSD for multiple comparisons after performing one way ANOVA). For Control n = 42, *dl NES S>A* n = 31, and for *dl NES S>D* n = 44. Embryos of a certain genotype were collected from the same cage either on the same day or subsequent days.

Table S1. A list of the *Drosophila melanogaster* lines, primers, plasmids, reagents, and software used in this study.

Available for download at
https://journals.biologists.com/dev/article-lookup/doi/10.1242/dev.202775#supplementary-data

Development • Supplementary information

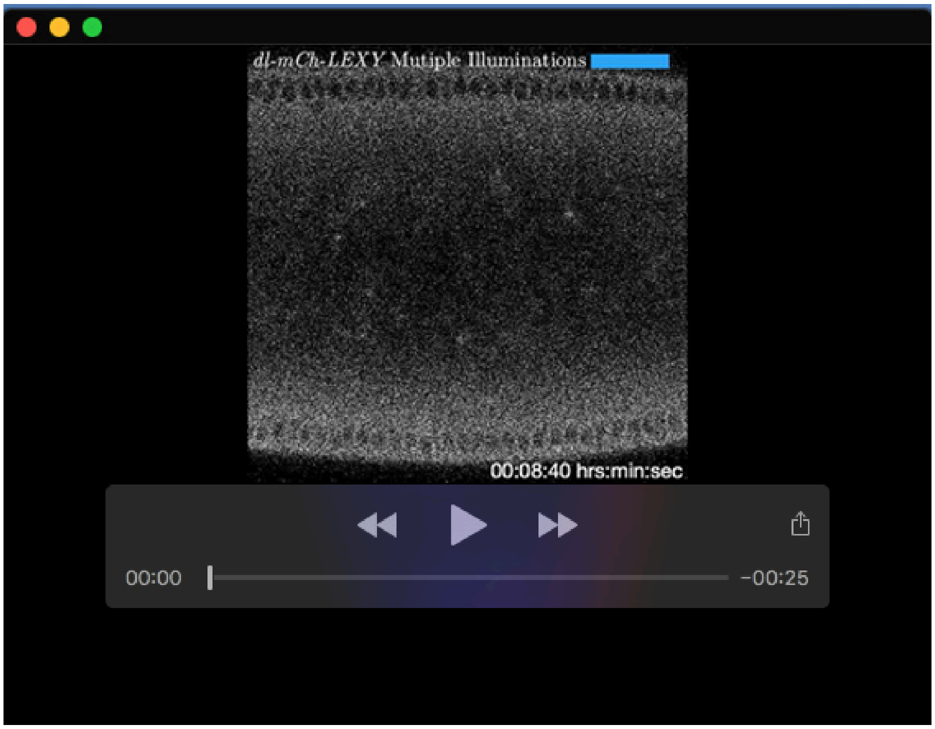

Movie 1. Blue light-induced export of DL-mCherry-LEXY is rapid and reversible. Related to Fig. 1. Blue bar in the upper right corner represents frames under blue light in this and all subsequent movies.

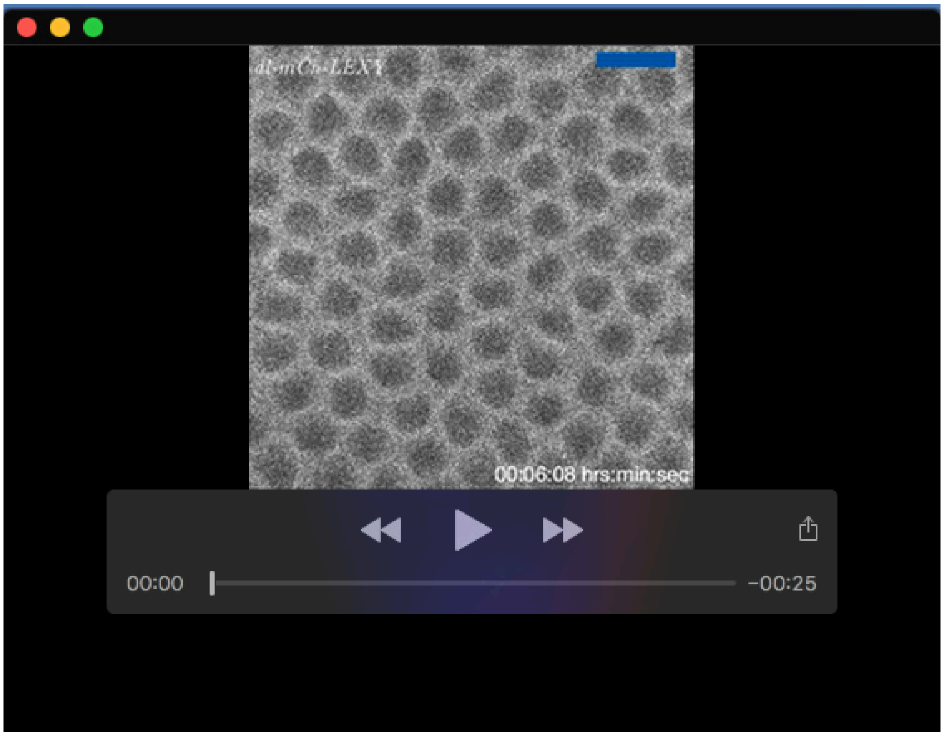

Movie 2. Recovery of DL-mCherry-LEXY after blue light. Related to Figure 1.

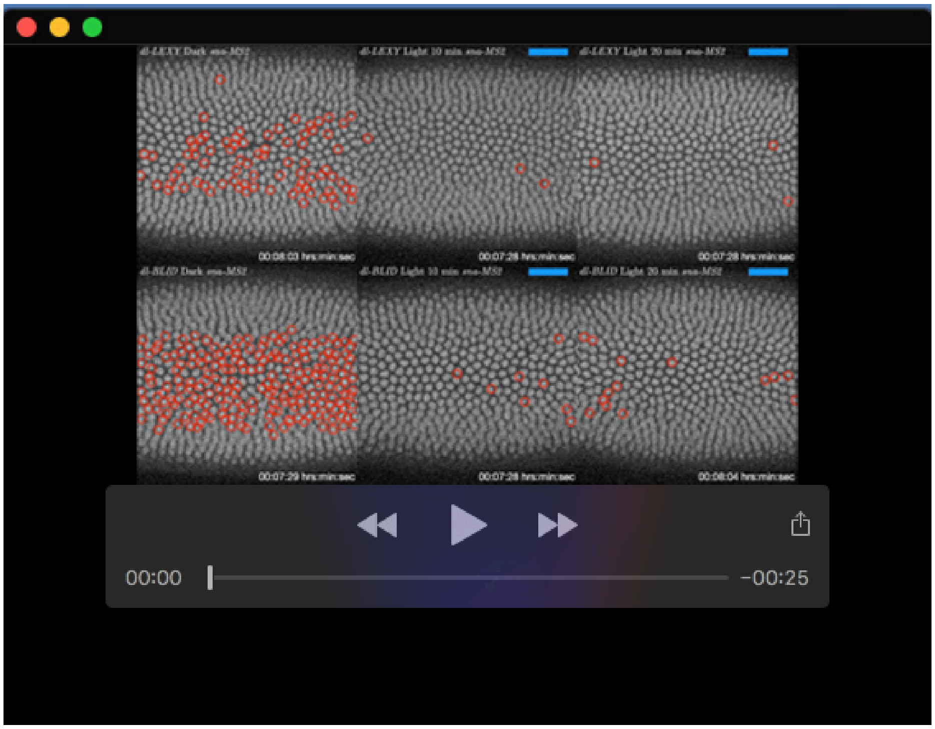

Movie 3. *sna-MS2* in *dl-LEXY* and *dl-BLID* in the dark, with 10 min of blue light, and with 20 min of blue light. Related to Fig. 2. Nascent transcription was only detected above a certain threshold and was marked by circles in this and all following movies.

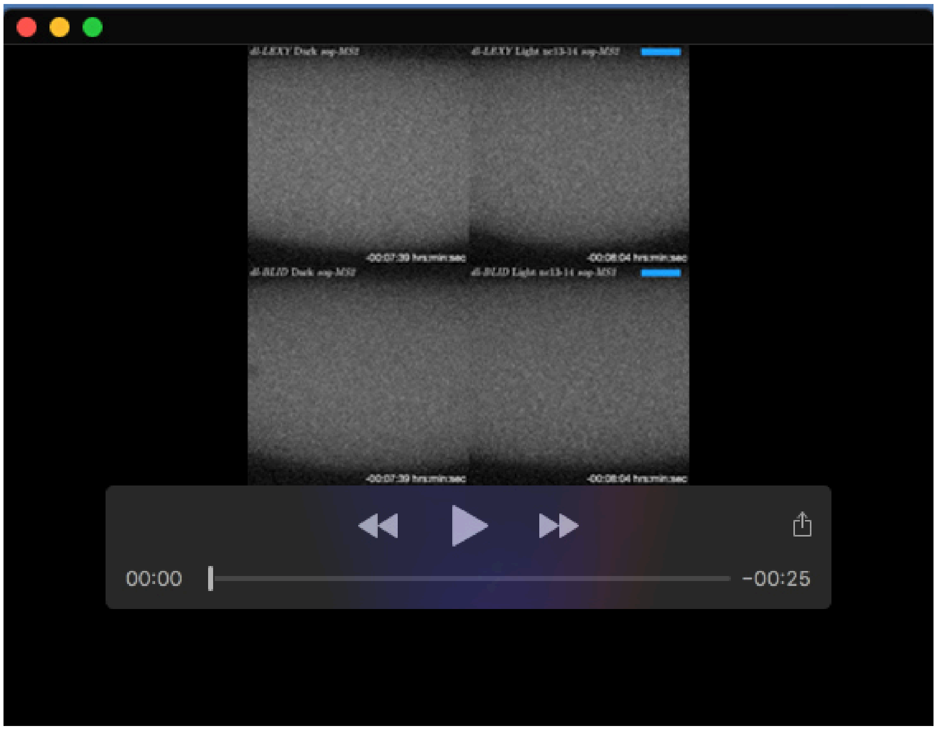

Movie 4. *sog-MS2* in *dl-LEXY* and *dl-BLID* in the dark and with blue light throughout nc13 and 14. Related to Fig. 3.

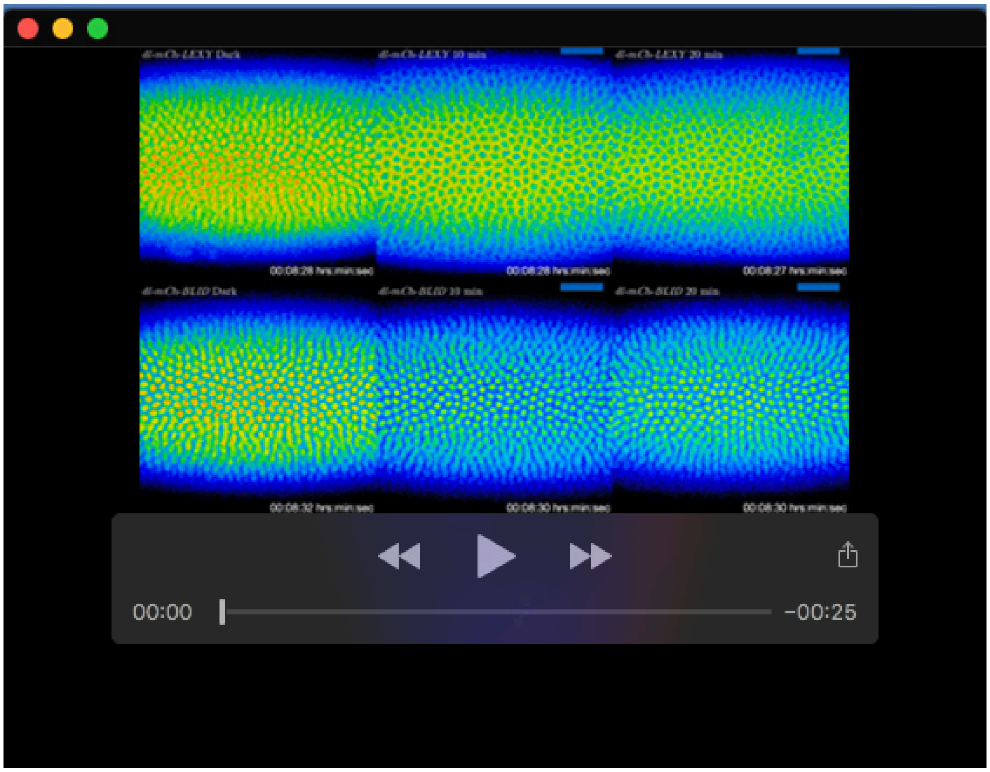

Movie 5. DL-mCh-LEXY and DL-mCh-BLID in the dark, with 10 min of blue light and with 20 min of blue light. Related to Fig. 4.

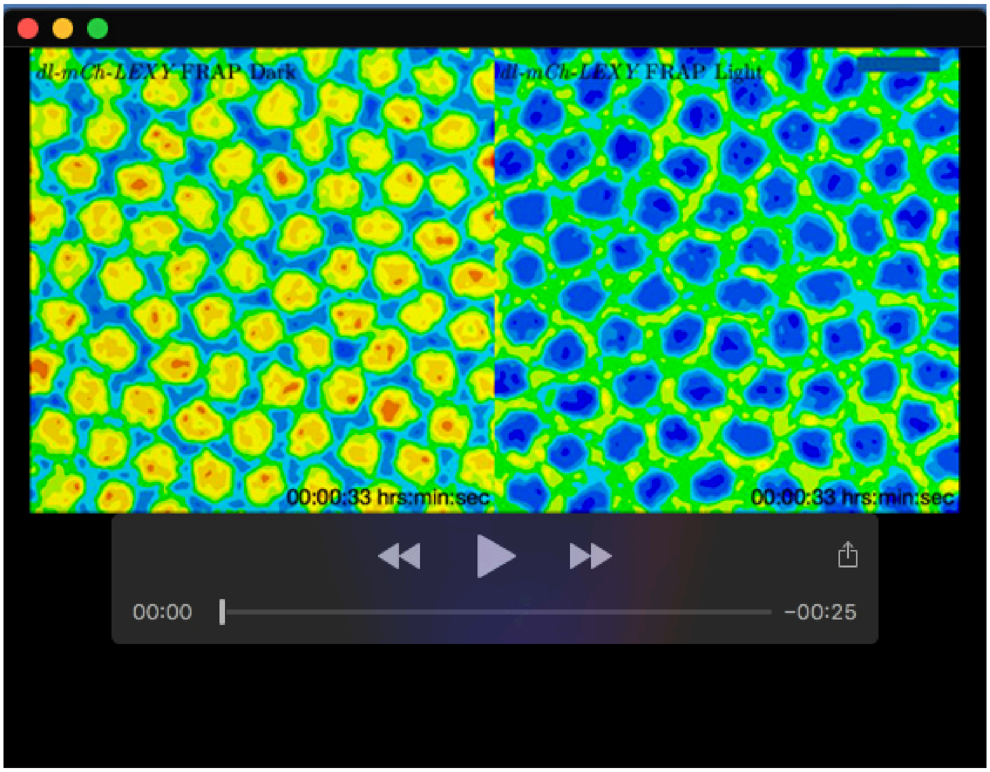

Movie 6. FRAP of DL-mCh-LEXY in an ROI in the dark and under blue light. Related to Fig. 5.

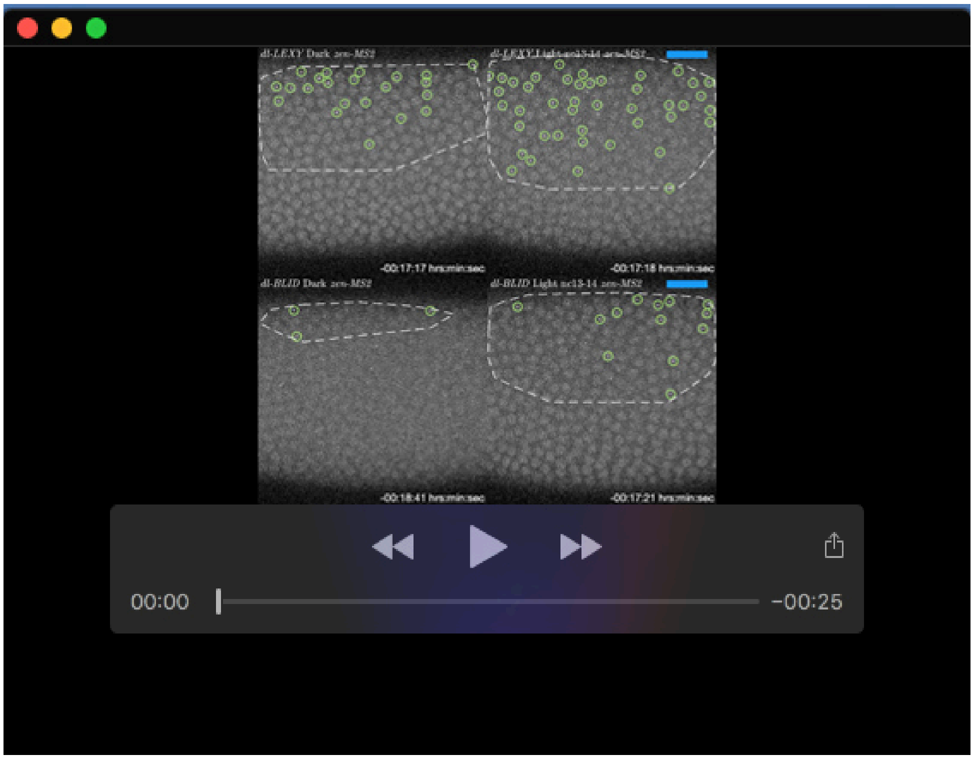

Movie 7. *zen-MS2* in *dl-LEXY* and *dl-BLID* in the dark and with blue light throughout nc13 and 14. Related to Fig. S3.

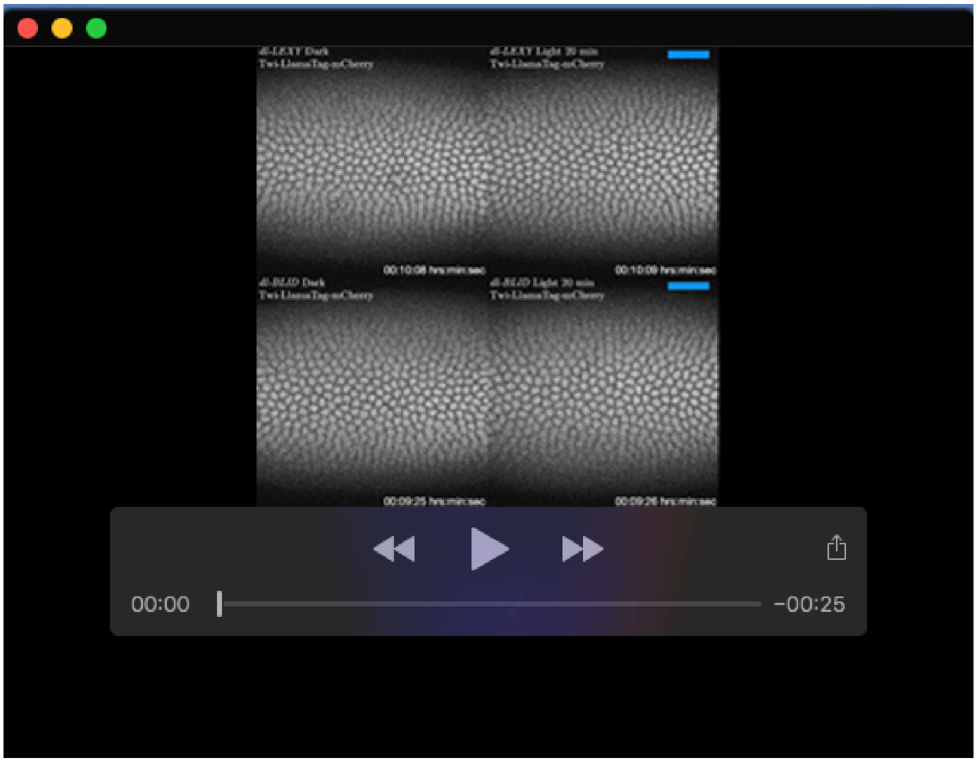

Movie 8. Twi-LlamaTag bound to mCherry with 10 min of blue light. Related to Fig. S4.

Development • Supplementary information
