# Supplementary material for: Target gene responses differ when transcription factor levels are acutely decreased by nuclear export versus degradation
Source: Development. 2024 Nov 8;151(21):dev202775. doi: 10.1242/dev.202775 (PMC11574349; doi:10.1242/dev.202775)
Supplement: Supplementary file 1 [file develop-151-202775_review_history.pdf]

Target gene responses differ when transcription factor levels are acutely decreased by nuclear export versus degradation

James McGehee and Angelike Stathopoulos
DOI: 10.1242/dev.202775

Editor: James Briscoe

Review timeline

Original submission:	10 February 2024
Editorial decision:	11 March 2024
First revision received:	12 August 2024
Editorial decision:	23 September 2024
Second revision received:	30 September 2024
Accepted:	4 October 2024

Original submission

First decision letter

MS ID#: DEVELOP/2024/202775

MS TITLE: Target gene responses differ when transcription factor levels are acutely decreased by nuclear export versus degradation

AUTHORS: James McGehee and Angelike Stathopoulos

I have now received all the referees' reports on the above manuscript, and have reached a decision. The referees' comments are appended below, or you can access them online: please go to BenchPress and click on the 'Manuscripts with Decisions' queue in the Author Area.

As you will see, the referees recognise the importance of your work and express considerable interest in your work, but have some significant criticisms and recommend a substantial revision of your manuscript before we can consider publication. Each of the referees has specific questions that should be addressed. Editing the text to improve clarity will answer some of these. In addition, providing quantitation of key results will be important. In particular, I agree with the referees that documenting the gradients of DL-LEXY and DL-BLID in the dark seems to be important. I would also direct your attention to the questions Referee 3 has about quantitation.

If you are able to revise the manuscript along the lines suggested, which may involve further experiments, I will be happy to receive a revised version of the manuscript. Your revised paper will be re-reviewed by one or more of the original referees, and acceptance of your manuscript will depend on your addressing satisfactorily the reviewers' major concerns. Please also note that Development will normally permit only one round of major revision. If it would be helpful, you are welcome to contact us to discuss your revision in greater detail. Please send us a point-by-point response indicating your plans for addressing the referees’ comments, and we will look over this and provide further guidance.

Please attend to all of the reviewers' comments and ensure that you clearly highlight all changes made in the revised manuscript. Please avoid using 'Tracked changes' in Word files as these are lost in PDF conversion. I should be grateful if you would also provide a point-by-point response detailing

© 2024. Published by The Company of Biologists under the terms of the Creative Commons Attribution License (https://creativecommons.org/licenses/by/4.0/).

how you have dealt with the points raised by the reviewers in the 'Response to Reviewers' box. If you do not agree with any of their criticisms or suggestions please explain clearly why this is so.

Reviewer 1

Advance summary and potential significance to field

This study represents a great studying into morphogen patterning in early fly embryos, employing cutting-edge, light-inducible technologies to dissect the temporal dynamics of the transcription factor Dorsal (DL) in patterning the dorsal-ventral axis of Drosophila embryos. By ingeniously utilizing two distinct light-inducible tags, DL-LEXY for nuclear export and DL-BLID for protein degradation, the authors have provided insights into how spatial and temporal regulation of DL influences the expression of its target genes, snail (sna) and short-gastrulation (sog), critical for embryonic development.
The differential effects of these light-inducible systems on sna and sog expression underscore the complexity of DL-mediated gene regulation. The observation that DL-LEXY leads to sna repression but retains sog expression contrasted with DL-BLID's preservation of sna and loss of sog, intriguingly highlights the nuanced role of DL localization and concentration in target gene activation. Moreover, the analysis of DL levels under blue light exposure offers a plausible explanation for these phenomena, emphasizing the importance of DL concentration and nuclear retention in the patterning process.
Additionally, the study elegantly addresses potential cofactor involvement and employs fluorescence recovery after photobleaching (FRAP) to investigate the kinetics of DL nuclear import and export. The finding that DL-LEXY can re-enter the nucleus even under conditions favoring export, and the proposal that continuous import-export dynamics contribute to the observed gene expression patterns, provide valuable insights into the mechanistic underpinnings of morphogen-mediated patterning.

Comments for the author

Here, I will outline some points/comments to be addressed:
1) Are the DL constructs fully viable? As this is young technology, it would be great to have this characterized further.
2) While the authors do a great job of exploring the dynamics with FRAP, it would be great if they could quantify the levels of DL in Figure 1.
Additionally, it would be ideal if they pushed into higher resolution or super-resolution within the nucleus. This can be done on fixed preps, so it should be straightforward.
3)For the data around figures 2/3, pending the point above, it could be great if the authors could explore the subnuclear DL localization around the sites of active transcription. This could be done off the MS2 spots, as per Tsai et al., 2022 (dev200266). It could be possible the differences could be between higher affinity enhancers (sog), that retain localized DL binding even under blue light conditions.
4) In figure 5, it's hard to see the measurement ROI in panel A.
5) In the discussion, it would be nice to have a paragraph discussing the transcription factor levels in a broader context. For example, Joanna Wysocka has done some interesting research on TF concentrations (and there are more).

In conclusion, this work not only sheds light on the sophisticated mechanisms governing Drosophila embryonic development, but also sets a precedent for using light-inducible technologies to study dynamic biological processes. The implications of these findings extend beyond Drosophila, offering potential insights into the general principles of morphogen-mediated tissue patterning in multicellular organisms.

Reviewer 2

Advance summary and potential significance to field

© 2024. Published by The Company of Biologists under the terms of the Creative Commons Attribution License (https://creativecommons.org/licenses/by/4.0/).

In the current manuscript the authors explore a topic of significant current interest in the field of development, namely how the dynamics of morphogen gradients shape the response of their target genes.
Specifically, the authors focus on the transcription factor Dorsal (DL), which patterns the dorso-ventral axis of the early Drosophila embryo. They dynamically modulate the DL nuclear concentration gradient using two different blue-light inducible approaches. The fist relies on the LEXY tag which induces rapid and reversible nuclear export of DL upon illumination. The second technique used the BLID tag which causes the Dorsal protein to degrade irreversibly on a slower timescale. By observing how target genes respond differently to the same durations of illumination in DL-LEXY versus DL-BLID embryos they draw several conclusions. The major ones are that a threshold model is insufficient to explain the differences they observe, and that the specific kinetics of DL import-export play a role in defining the expression of its target genes.

I believe the reagents generated in this paper will be useful to many, and that the topic is of broad interest.
However, there are unfortunately significant issues which mean the data presented does not justify the conclusions draw. This manuscript would need to be substantially revised before it would be ready to publish in Development.

Comments for the author

Major Issues:

1) Many of the conclusions are underpinned by the assumption that DL-LEXY and DL-BLID form equivalent gradients and that the proteins have similar properties in the dark. However, this is not consistent with the very different phenotypes of these lines. In this study the authors find that DL-mCh-LEXY flies are “homozygous viable, fertile and healthy” while they previously reported that the DL-mCh-BLID fusions are severely compromised and “do not retain Dl function” (Irizarry et al., 2020). The DL-BLID fusion that lacks mCherry partially rescues, but only at lower temperatures and with substantially reduced viability of less that 50%. Even in the dark the DL-BLID embryos often had severe patterning defects in both snail and sog expression. This again is quite different to DL-BLID which is “homozygous viable, fertile and healthy”.
If the authors wish to press the point that different behavior of target genes is due to differential kinetics of import-export they need to rule out more obvious explanations like changes to the gradient shape or amplitude (which is not quantified for either construct) or protein activity, which would explain the different phenotypes. The different phenotypes of the constructs also need to be clearly stated and discussed in the text.

2) There is contradicting data presented when comparing snail transcription in the dark in the DL-LEXY and DL-BLID contexts. Fig S2 shows that the mean number of sna-MS2 spots in mid to late nc14 in the dark is the same for DL-LEXY and DL-BLID contexts, with 9 embryos measured for each background. Yet Figures 2K and 2L show the opposite, with DL-BLID having double the number of sna-MS2 spots as DL-LEXY during this period in the dark, 3 embryos each. This two-fold difference is also far outside the error so not a fluke.
This contradiction needs to be reconciled. By looking at the shape of the snail pattern in the images and going through the methods I expect this has to do with having a range of different AP positions, which does not seem to be controlled for. Snail is narrower and less robustly expressed near the cephalic furrow where DL concentrations are lower.

3) Even though one of the main conclusions is about how target genes respond to different DL levels nuclear DL levels are not quantified. The images in Fig 4 are not sufficient to determine by eye what the DL concentration is in either construct or how it changes in time.
The nuclear concentration dynamics of of DL in the DL-LEXY and DL-BLID backgrounds need to be quantified and graphed.

4) An alternative explanation for snail’s differential behavior would have to do with twist acting like a “molecular ratchet”. According to the authors’ 2020 study “once Twi reaches sufficient levels to support its own auto-activation, Dl is no longer required to support sna expression”. The slower decrease in DL-BLID during illumination would allow Twi expression to become established allowing snail to be expressed.

© 2024. Published by The Company of Biologists under the terms of the Creative Commons Attribution License (https://creativecommons.org/licenses/by/4.0/).

However, the rapid and sustained drop in DL-LEXY levels would likely compromise the establishment of Twi expression which is necessary for snail expression late.
This explanation could be tested using the Twist protein reporter or should at the very least be discussed as an alternative explanation.

Minor Issues

1) On page 54 in the recombinant DNA table a twi-MS2 donor plasmid is mentioned which I could not find used in this study.

2) The dl-mCh-BLID fly line does not apear in the reagents table.

3) Multiple other groups have published papers using LEXY to control transcription factor concentration in the fly embryo in recent years (e.g. Gregor, Garcia and Furlong), these studies should be cited.

Reviewer 3

Advance summary and potential significance to field

In this paper, the authors investigate the response of target genes to changes in Dorsal, a transcription factor morphogen in the early Drosophila embryo. They use two optogenetic approaches – one to degrade and one to remove Dorsal from the nucleus – to spatiotemporally perturb Dorsal. By comparing differences in the downstream gene response to perturbations, they conclude that Dorsal readout – especially for snail – is dependent on Dorsal kinetics, and not just local absolute concentration levels.

Overall, the question being asked is interesting and an important one. There has been a lot of work recently exploring the temporal readout of morphogens. Such a direct comparison of mechanisms as provided here is an important addition to the field.

Having said that, the paper requires significant work to bring to the standard I would expect at Development. In particular, the conclusions seem to be drawn from only a qualitative analysis of the results. This should be rectifiable through application of suitable approaches.

Comments for the author

Major concerns:

1. The abstract is very convoluted in its writing. The main conclusions are difficult to parse, with too many acronyms. I suggest rewriting, focusing on describing the type of perturbation (e.g., degradation) and observed phenotype rather than the specific constructs.

2. The quantification is currently very limited. For example, figure 4 shows heat maps for a single embryo. Particularly when comparing more subtle changes (e.g., 10 vs 20 min LEXY), a clear quantification across multiple embryos is needed.

Relatedly, there are often comments such as “we did not notice any obvious changes in sog or zen expression”. Yet, as is well known, careful quantification in the early embryo can reveal important insights into the gene regulation and morphogen readout (e.g., work from Gregor lab).

Another example is the statement regarding photobleaching in Figure 4. A careful analysis across multiple embryos can help reveal whether there are more complicated dynamics.

In summary, the current confidence of statements regarding the readout of Dorsal are not supported by sufficiently careful analysis of the data. The authors may well be correct, but more work is needed to justify such statements.

© 2024. Published by The Company of Biologists under the terms of the Creative Commons Attribution License (https://creativecommons.org/licenses/by/4.0/).

3. In Figure 2K, the 10min condition for dl-LEXY recovers to far above the Dark condition. This is very surprising. What explanation is there for such behavior?

4. The FRAP experiment is underdeveloped. The data shown in 5C-D shows that the signal remains in the internuclear domain during recovery, so unclear to me how the authors justify the statement "…transits in and out…".. There is a close-up in Figure 5B but this is only briefly discussed in the main paper. There are two time-scales (during and after blue light). These need to be carefully distinguished. No recovery curve fitting is provided to provide estimation of timescales.

Further, the FRAP experiment is too coarse. A bleaching of just one nucleus would be much better. Then, the actual nucleus recovery can be more clearly distinguished from simply recovery into a larger domain, which includes multiple nuclei.

Finally, it is claimed the experiment is repeated three times but no quantification or analysis of reproducibility. (It may be this figure was altered late, as it is mis-referenced as figure 3I-K in the Discussion).

5. Figure 6 is potentially a very strong addition. Yet, again, it only gives a flavor of the data. Strong conclusions are stated from only qualitative panels.
This needs to be revisited and analyzed in a more detailed fashion.

6. What evidence is there that expression levels are same between all conditions in the dark?

7. While the Discussion of the results is good and clear, Figure 7 is too convoluted. It needs better labelling and direction – the addition of a cartoon is good, but the current one requires too much reader interpretation to be helpful.

Minor points:

1. "…(e.g. Bothma et al. 2015)" – odd to use e.g. here. Further, this paper was published with another paper on the same system from the Dostatni lab. It is incorrect to only select one for referencing.
2. Figure 2I-K – there seems to be a small recovery in sna-MS2 dl-LEXY after 20 minutes. Is this completely repressed if a longer time window of blue light illumination?
3. Do not use s.e.m. without compelling reason. Here, with such a low number of experiments, s.e.m. is clearly not justified.
4. There is no Fig. 6F but referenced in text.

First revision

<u>Author response to reviewers' comments</u>

***** Reviewer 1 Advance Summary and Potential Significance to Field:
This study represents a great studying into morphogen patterning in early fly embryos, employing cutting-edge, light-inducible technologies to dissect the temporal dynamics of the transcription factor Dorsal (DL) in patterning the dorsal-ventral axis of Drosophila embryos. By ingeniously utilizing two distinct light-inducible tags, DL-LEXY for nuclear export and DL-BLID for protein degradation, the authors have provided insights into how spatial and temporal regulation of DL influences the expression of its target genes, snail (sna) and short-gastrulation (sog), critical for embryonic development.

The differential effects of these light-inducible systems on sna and sog expression underscore the complexity of DL-mediated gene regulation. The observation that DL-LEXY leads to sna repression but retains sog expression, contrasted with DL-BLID's preservation of sna and loss of sog, intriguingly highlights the nuanced role of DL localization and concentration in target gene activation. Moreover, the analysis of DL levels under blue light exposure offers a plausible

© 2024. Published by The Company of Biologists under the terms of the Creative Commons Attribution License (https://creativecommons.org/licenses/by/4.0/).

explanation for these phenomena, emphasizing the importance of DL concentration and nuclear retention in the patterning process.Additionally, the study elegantly addresses potential cofactor involvement and employs fluorescence recovery after photobleaching (FRAP) to investigate the kinetics of DL nuclear import and export. The finding that DL-LEXY can re-enter the nucleus even under conditions favoring export, and the proposal that continuous import-export dynamics contribute to the observed gene expression patterns, provide valuable insights into the mechanistic underpinnings of morphogen-mediated patterning.

Reviewer 1 Comments for the Author:
Here, I will outline some points/comments to be addressed:
1) Are the DL constructs fully viable? As this is young technology, it would be great to have this characterized further.
We agree this is a useful metric and tried to do viability/hatching assays in both the dark and light for a control, DL-LEXY, and DL-BLID. However, because these lines contained balancers, which resulted in 50% reduced viability alone, further effects on viability made it difficult to detect a difference in viability between dark and light. To address this, we instead measured the width of the *sna* domain in fixed embryos. While we detected a decrease in *sna* width in DL-LEXY and DL-BLID, it was not significantly different from control. These data were added to supplementary figure 1. In addition, we added panels to figure 1 that show that DL-LEXY embryos exposed to blue light in early development fail to undergo gastrulation. Assay of *sna* expression and gastrulation phenotypes are direct readouts of Dorsal activity level, whereas viability/hatching assays would be indirect.

2) While the authors do a great job of exploring the dynamics with FRAP, it would be great if they could quantify the levels of DL in Figure 1. Additionally, it would be ideal if they pushed into higher resolution or super-resolution within the nucleus. This can be done on fixed preps, so it should be straightforward.
We now quantify the levels of DL and display it as part of Figure 1.
For the second point, we feel super-resolution imaging within the nucleus is beyond the scope of this paper and would be better suited for a future study. It's a very interesting suggestion and an area we would like to follow-up, perhaps in collaboration with other experts.

3) For the data around figures 2/3, pending the point above, it could be great if the authors could explore the subnuclear DL localization around the sites of active transcription. This could be done off the MS2 spots, as per Tsai et al., 2022 (dev200266). It could be possible the differences could be between higher affinity enhancers (sog), that retain localized DL binding even under blue light conditions.
As discussed in point 2 above - while examining DL subnuclear localization is an interesting idea, we believe this would be better suited for a future study. Our blue light illumination scheme is more amenable to live imaging than fixed imaging. Thus while we think subnuclear localization is an important area for study, we think it is better suited for future studies that carefully focus on this question.

4) In figure 5, it's hard to see the measurement ROI in panel A.
We apologize for not making the measurement ROI more visible. In any case, we have refined our image processing pipeline, and now the measurement ROI is no longer needed, so it has been removed. Instead the nuclei are segmented automatically and the center nucleus is tracked over time

5) In the discussion, it would be nice to have a paragraph discussing the transcription factor levels in a broader context. For example, Joanna Wysocka has done some interesting research on TF concentrations (and there are more).
We would be happy to expand the discussion but due to the length restriction at *Development*, and the need to make sure the results are clear - including addressing reviewers' concerns - we unfortunately do not have much space. We did add a few sentences to the discussion on this suggested topic and now include a reference to Naqvi et al. 2023 from the Wysocka lab.

In conclusion, this work not only sheds light on the sophisticated mechanisms governing Drosophila embryonic development, but also sets a precedent for using light-inducible technologies to study dynamic biological processes. The implications of these findings extend beyond Drosophila, offering

© 2024. Published by The Company of Biologists under the terms of the Creative Commons Attribution License (https://creativecommons.org/licenses/by/4.0/).

potential insights into the general principles of morphogen-mediated tissue patterning in multicellular organisms.
We appreciate the positive feedback. Thank you!

***** Reviewer 2 Advance Summary and Potential Significance to Field:
In the current manuscript the authors explore a topic of significant current interest in the field of development, namely how the dynamics of morphogen gradients shape the response of their target genes. Specifically, the authors focus on the transcription factor Dorsal (DL), which patterns the dorso-ventral axis of the early Drosophila embryo. They dynamically modulate the DL nuclear concentration gradient using two different blue-light inducible approaches. The fist relies on the LEXY tag which induces rapid and reversible nuclear export of DL upon illumination. The second technique used the BLID tag which causes the Dorsal protein to degrade irreversibly on a slower timescale. By observing how target genes respond differently to the same durations of illumination in DL-LEXY versus DL-BLID embryos they draw several conclusions. The major ones are that a threshold model is insufficient to explain the differences they observe, and that the specific kinetics of DL import-export play a role in defining the expression of its target genes.
I believe the reagents generated in this paper will be useful to many, and that the topic is of broad interest. However, there are unfortunately significant issues which mean the data presented does not justify the conclusions draw. This manuscript would need to be substantially revised before it would be ready to publish in Development.

Reviewer 2 Comments for the Author:
Major Issues:

1) Many of the conclusions are underpinned by the assumption that DL-LEXY and DL-BLID form equivalent gradients and that the proteins have similar properties in the dark. However, this is not consistent with the very different phenotypes of these lines. In this study the authors find that DL-mCh-LEXY flies are “homozygous viable, fertile and healthy” while they previously reported that the DL-mCh-BLID fusions are severely compromised and “do not retain Dl function” (Irizarry et al., 2020). The DL-BLID fusion that lacks mCherry partially rescues, but only at lower temperatures and with substantially reduced viability of less that 50%. Even in the dark the DL-BLID embryos often had severe patterning defects in both snail and sog expression. This again is quite different to DL-BLID [sic DL-LEXY] which is “homozygous viable, fertile and healthy”.
We interpret this comment as if DL-LEXY is more viable than DL-BLID could this explain the differences in target gene expression? Under conditions of extended blue light at nc14, we emphasize that *sna* is retained in DL-BLID (the stock that requires low temperature with substantially reduced viability) but *sna* is not present in DL-LEXY (the stock that is healthier).

If the authors wish to press the point that different behavior of target genes is due to differential kinetics of import-export they need to rule out more obvious explanations like changes to the gradient shape or amplitude (which is not quantified for either construct) or protein activity, which would explain the different phenotypes. The different phenotypes of the constructs also need to be clearly stated and discussed in the text.
To address this point, we have measured the width of the *sna* domain in DL-LEXY and DL-BLID in the dark (Fig. S1). We found a slight decrease in the width in DL-BLID, but this was not statistically significant. We also measured the DL gradient in *dl-LEXY* and *dl-BLID* in the dark and compared them to *yw*. We found that the peak levels in *dl-BLID* are reduced in the dark. However, since *dl-BLID* is the line that supports *sna* with 20 min of blue light, this difference cannot explain why *dl-LEXY* does not support *sna* expression with 20 min of blue light We also measured the DL levels in Figure 4 for both *dl-mCh-LEXY* and *dl-mCh-BLID*. We found that the levels of DL are lower in BLID than they are in LEXY in the dark (as well as upon blue light illumination). Again, since BLID has lower DL levels and is the line that retains *sna*, differences in the dark condition are an insufficient explanation.

2) There is contradicting data presented when comparing snail transcription in the dark in the DL-LEXY and DL-BLID contexts. Fig S2 shows that the mean number of sna-MS2 spots in mid to late nc14 in the dark is the same for DL-LEXY and DL-BLID contexts, with 9 embryos measured for each background. Yet Figures 2K and 2L show the opposite, with DL-BLID having double the number of sna-MS2 spots as DL-LEXY during this period in the dark, 3 embryos each. This two-fold difference is also far outside the error so not a fluke.This contradiction needs to be reconciled. By looking at

© 2024. Published by The Company of Biologists under the terms of the Creative Commons Attribution License (https://creativecommons.org/licenses/by/4.0/).

the shape of the snail pattern in the images and going through the methods I expect this has to do with having a range of different AP positions, which does not seem to be controlled for. Snail is narrower and less robustly expressed near the cephalic furrow where DL concentrations are lower.
We have updated the text to clarify how we generated the plot in Figure S2B, which we will also summarize here - as follows. For all conditions (dark, light 10 min, light 20 min) the embryo is kept in the dark for ~2 min before the illumination begins. Thus, we took the starting number of spots/transcription sites of all 19 traces from Fig 2K and 2L and plotted them in Fig S2. These data demonstrate the variability in sna-MS2 spot number associated with either DL-LEXY or DL-BLID *in the dark* (as even embryos subsequently exposed to light are initially in the dark).

We then performed a statistical test between the various conditions. We found there is no statistical difference in the starting number of transcription sites between any condition.
To help clarify this point we color coded the points in Fig S2B to show how they relate to Fig 2K and 2L. To the best of our ability, we shield the embryos from light, but even brief exposures/leaks could contribute to this observed variability in starting *sna* levels. The helices (BLID and LEXY) could also be spontaneously active at some low level and contribute to this variability.

The field of view was positioned at roughly the center (50%) of the embryo by eye using transmitted light (filtered for only red light to protect the embryo from blue light that activates LEXY and BLID). The position was then adjusted slightly to center the signal around the highest point of the embryo, which should be close to the middle of the embryo. This procedure was performed similarly for all embryos. We also checked the field of view by zooming out on embryos that had undergone gastrulation and find that the cephalic furrow region is not included in our field of view, which is located more in the center of the trunk.

We repeated the experiment and took an additional 3 movies for each condition and observed the same trend. All of these data were pooled together for Figure 2.

3) Even though one of the main conclusions is about how target genes respond to different DL levels, nuclear DL levels are not quantified. The images in Fig 4 are not sufficient to determine by eye what the DL concentration is in either construct or how it changes in time. The nuclear concentration dynamics of of DL in the DL-LEXY and DL-BLID backgrounds need to be quantified and graphed.
To address this issue, we quantified DL levels in the images in Fig 4 and added these plots to that figure.

4) An alternative explanation for snail's differential behavior would have to do with twist acting like a "molecular ratchet". According to the authors' 2020 study "once Twi reaches sufficient levels to support its own auto-activation, Dl is no longer required to support sna expression". The slower decrease in DL-BLID during illumination would allow Twi expression to become established allowing snail to be expressed. However, the rapid and sustained drop in DL-LEXY levels would likely compromise the establishment of Twi expression which is necessary for snail expression late. This explanation could be tested using the Twist protein reporter or should at the very least be discussed as an alternative explanation.
To address this, we imageed the Twi-LlamaTag under 20 min of blue light as in Figures 2 and 4, in DL-LEXY and DL-BLID, and compared the levels of Twi in these lines. We found that the blue light does not affect Twi levels and show this in Fig. S4.

Minor Issues

1) On page 54 in the recombinant DNA table a twi-MS2 donor plasmid is mentioned which I could not find used in this study.
We removed the twi-MS2 plasmid from the table as it was not used in this study.

2) The dl-mCh-BLID fly line does not apear in the reagents table.
We apologize for the oversight and have added the dl-mCh-BLID fly line to the table. It was published in Irizarry et al. *Genes Dev* 2020.

© 2024. Published by The Company of Biologists under the terms of the Creative Commons Attribution License (https://creativecommons.org/licenses/by/4.0/).

3) Multiple other groups have published papers using LEXY to control transcription factor concentration in the fly embryo in recent years (e.g. Gregor, Garcia and Furlong), these studies should be cited.
We had cited Furlong (Kögler et al., 2021) and Gregor (Singh et al., 2022). We now also cite Garcia's paper on LEXY, a preprint on bioarchive.

***** Reviewer 3 Advance Summary and Potential Significance to Field:
In this paper, the authors investigate the response of target genes to changes in Dorsal, a transcription factor morphogen in the early Drosophila embryo. They use two optogenetic approaches - one to degrade and one to remove Dorsal from the nucleus - to spatiotemporally perturb Dorsal. By comparing differences in the downstream gene response to perturbations, they conclude that Dorsal readout - especially for snail - is dependent on Dorsal kinetics, and not just local absolute concentration levels.

Overall, the question being asked is interesting and an important one. There has been a lot of work recently exploring the temporal readout of morphogens. Such a direct comparison of mechanisms as provided here is an important addition to the field.

Having said that, the paper requires significant work to bring to the standard I would expect at Development. In particular, the conclusions seem to be drawn from only a qualitative analysis of the results. This should be rectifiable through application of suitable approaches.

Reviewer 3 Comments for the Author:
Major concerns:

1. The abstract is very convoluted in its writing. The main conclusions are difficult to parse, with too many acronyms. I suggest rewriting, focusing on describing the type of perturbation (e.g., degradation) and observed phenotype, rather than the specific constructs.
We have rewritten the abstract in an attempt to make it simpler and clearer, avoiding the use of acronyms, and using a description of the perturbation and the observed phenotype as suggested.

2. The quantification is currently very limited. For example, figure 4 shows heat maps for a single embryo. Particularly when comparing more subtle changes (e.g., 10 vs 20 min LEXY), a clear quantification across multiple embryos is needed.
As mentioned in the response to reviewer two above (major comment three), we quantified the levels in these movies and now display the plots at the bottom of Figure 4.

Relatedly, there are often comments such as "we did not notice any obvious changes in sog or zen expression". Yet, as is well known, careful quantification in the early embryo can reveal important insights into the gene regulation and morphogen readout (e.g., work from Gregor lab).
We agree that a careful analysis of the bursting dynamics, as commonly used by the Gregor lab, might reveal changes in the dark vs the light, however, we think these merit careful studies of their own. Here in the current study, we sought to shift the boundaries of target genes through the reduction of nuclear DL. Thus, we quantified the domains and did not observe differences.

Another example is the statement regarding photobleaching in Figure 4. A careful analysis across multiple embryos can help reveal whether there are more complicated dynamics.
We quantified the levels of nuclear DL in LEXY and BLID in Figure 4 and displayed the plots. We have also removed the noted comment from the text.

In summary, the current confidence of statements regarding the readout of Dorsal are not supported by sufficiently careful analysis of the data. The authors may well be correct, but more work is needed to justify such statements.
We hope that the changes above have addressed the reviewer's concerns.

3. In Figure 2K, the 10min condition for dl-LEXY recovers to far above the Dark condition. This is very surprising. What explanation is there for such behavior?
To attempt to address this, we plotted the number of spots normalized by the initial number of spots to determine if it was affected by larger or smaller initial domains. The levels of the 10 min condition still go above the dark condition, however, there is a lot of variability in the final level.

© 2024. Published by The Company of Biologists under the terms of the Creative Commons Attribution License (https://creativecommons.org/licenses/by/4.0/).

We do not have a verified explanation for this observation. We can speculate that *sna* may detect a change in DL such that the recovery reaches a higher level, or that due to Sna autoregulation, there is a decrease in the repressor and when DL returns *sna* expression overshoots because of the reduction in repressor levels. While this is an interesting phenomenon, we believe further investigation would be better suited for a future study.

4. The FRAP experiment is underdeveloped. The data shown in 5C-D shows that the signal remains in the internuclear domain during recovery, so unclear to me how the authors justify the statement "…transits in and out…".. There is a close-up in Figure 5B but this is only briefly discussed in the main paper. There are two time-scales (during and after blue light). These need to be carefully distinguished. No recovery curve fitting is provided to provide estimation of timescales.
To address this, we now show magnified views of the bleached area at various time points - before, during, and after the bleach. We perform image segmentation on the nuclei and track the nucleus at the center of the bleached region. Only this nucleus is quantified and no internuclear regions are included. If at any time point the nucleus is not detectable we use the segmentation from the previous time point to measure intensity. The image segmentation was checked manually. We now conduct recovery curve fitting and provide estimates of timescales. We have moved analysis of the timescale for recovery from blue light illumination to figure 1. For comparison, we also perform FRAP and analysis on *dl-LEXY* embryos in the dark.

Further, the FRAP experiment is too coarse. A bleaching of just one nucleus would be much better. Then, the actual nucleus recovery can be more clearly distinguished from simply recovery into a larger domain, which includes multiple nuclei.
We updated the text to make it clear that we only quantified the levels of a single nucleus, the center nucleus in the bleached ROI. We apologize that it was hard to see the yellow spot that marked the single nucleus. In the revised version, we no longer need to use a ROI for quantification and instead track a single nucleus (see point above for a discussion of how we performed image segmentation on this nucleus). We attempted to bleach only a single nucleus, however we found it difficult to actually maintain low levels in the nucleus by bleaching a smaller ROI within a single nucleus. Thus, we decided to bleach a larger area and repeated this three times. We fit a single exponential to the recovery and have reported the parameter estimates. We also added a comparison between light and dark conditions. While we agree with the idea that the estimated recovery is affected by the larger bleach area, we think that with the dark control it is now clear that the recovery rates are different. We expect that the recovery rates would be faster if we only bleached a single nucleus by eliminating the contribution of diffusion. However, the observation that it was difficult to bleach a single nucleus under blue light only further supports the idea that the recovery under blue light is more rapid than in the dark. In addition, one might hypothesize that part of the recovery is due to diffusion and that the cytoplasmic availability of Dorsal is different in the dark versus the light (since export under blue light releases more Dorsal into the cytoplasm) and this will affect diffusion's contribution to the recovery rate. While we think this is likely true, we also think this increased recovery due to diffusion results in greater flux of Dorsal in and out of the nucleus and that this is what disrupts *sna* activation.

Finally, it is claimed the experiment is repeated three times but no quantification or analysis of reproducibility. (It may be this figure was altered late, as it is mis-referenced as figure 3I-K in the Discussion).
We have plotted the recovery curves for the three replicates and show the recovery curve fitting as well. Thank you for pointing out the improper figure call, which we have fixed.

5. Figure 6 is potentially a very strong addition. Yet, again, it only gives a flavor of the data. Strong conclusions are stated from only qualitative panels. This needs to be revisited and analyzed in a more detailed fashion.
We quantified the levels of nuclear DL in the supplement, and quantified the expression domain of the in situ images, which are included in the main figure 6.

6. What evidence is there that expression levels are same between all conditions in the dark?
We added an analysis of *sna* expression in LEXY and BLID to supplementary figure 1. We did not observe a difference between LEXY and BLID.

© 2024. Published by The Company of Biologists under the terms of the Creative Commons Attribution License (https://creativecommons.org/licenses/by/4.0/).

7. While the Discussion of the results is good and clear, Figure 7 is too convoluted. It needs better labelling and direction – the addition of a cartoon is good, but the current one requires too much reader interpretation to be helpful.
We appreciate the positive feedback regarding the Discussion. We updated Figure 7 and hope it is now more clear.

Minor points:
1. "…(e.g. Bothma et al. 2015)" – odd to use e.g. here. Further, this paper was published with another paper on the same system from the Dostatni lab. It is incorrect to only select one for referencing.
The Bothma reference was a newer study that used MS2 to study *sna* for the first time. We now cite Garcia et al. 2013 and Lucas et al. 2013 that originally developed the MS2 for studying nascent transcription in *Drosophila* embryos.

2. Figure 2I-K – there seems to be a small recovery in sna-MS2 dl-LEXY after 20 minutes. Is this completely repressed if a longer time window of blue light illumination?
The blue light remains on during this time, even during the time when *sna-MS2* exhibits the slight recovery. It is possible that even in the presence of *dl-LEXY* that eventually some Twi is made that is able to support a very weak *sna* recovery. We have not imaged longer than 20 min from mid nc14 as the embryos typically begin to gastrulate shortly after this.

3. Do not use s.e.m. without compelling reason. Here, with such a low number of experiments, s.e.m. is clearly not justified.
We now show mean ± s.d for all graphs or plot the individual data.

4. There is no Fig. 6F but referenced in text. Corrected.

<u>Second decision letter</u>

MS ID#: DEVELOP/2024/202775

MS TITLE: Target gene responses differ when transcription factor levels are acutely decreased by nuclear export versus degradation

AUTHORS: James McGehee and Angelike Stathopoulos

Apologies for the delay in getting back to you. It took some time to receive the referees' reports and I then wanted to consult on the differences of opinion between the referees. The referees' comments are appended below, or you can access them online: please go to BenchPress and click on the 'Manuscripts with Decisions' queue in the Author Area.

The referees continue to express interest in your work, but Referee 2 raises significant concerns about the interpretation of the data. Referee 2 is concerned about apparentbaseline differences between DL-LEXY and DL-BLID in the absence of light and the effect this could have. Referee 2 suggests that the difference in Snail expression in DL-LEXY and DL-BLID could be explained by these baseline differences resulting in the expression of a low/medium threshold neurogenic repressor of snail. Given the data, this seems a plausible alternative explanation that, if correct, would change the conclusions of the paper.I would like to give you the opportunity to respond to this issue. Please consider whetherindirect effects from Dorsal-dependent neurogenic repressors could explain the data, and compare this to the proposed mechanism. To strengthen the conclusion about Dorsal nuclear import-export kinetics determining target gene expression, any additional evidence that rules out indirect effects would be valuable.

I will be happy receive a revised version of the manuscript that addresses this issue. Your revised paper will be re-reviewed by one or more of the original referees, and acceptance of your manuscript will depend on your addressing satisfactorily the reviewers' major concerns. If it would

© 2024. Published by The Company of Biologists under the terms of the Creative Commons Attribution License (https://creativecommons.org/licenses/by/4.0/).

be helpful, you are welcome to contact us to discuss your revision in greater detail. Please send us a point-by-point response indicating your plans for addressing the referees' comments, and we will look over this and provide further guidance.

Please attend to all of the reviewers' comments and ensure that you clearly highlight all changes made in the revised manuscript. Please avoid using 'Tracked changes' in Word files as these are lost in PDF conversion. I should be grateful if you would also provide a point-by-point response detailing how you have dealt with the points raised by the reviewers in the 'Response to Reviewers' box. If you do not agree with any of their criticisms or suggestions please explain clearly why this is so.

Reviewer 1

Advance summary and potential significance to field

The paper by McGehee and Stathopoulos presents an advance in understanding how transcription factor dynamics impact gene expression during Drosophila embryonic development, specifically focusing on the Dorsal (DL) transcription factor. The researchers utilized optogenetic tools to perturb DL levels through two different methods: nuclear export (using the LEXY system) and degradation (using BLID). Their experiments revealed that different perturbation methods, such as snail and short gastrulation (sog), produce distinct effects on target gene expression. Nuclear export of DL resulted in the loss of snail expression whereas degradation allowed snail expression to be retained. Additionally, sog expression was supported under nuclear export conditions but repressed under degradation, showing that the concentration and retention of DL in the nucleus affect gene regulatory outcomes.

This research has the potential to reshape current models of morphogen action by highlighting the importance of transcription factor kinetics, such as nuclear import-export dynamics, rather than relying solely on concentration thresholds.
By offering new insights into gene expression regulation during development, the study may influence broader research into morphogenetic patterning and transcriptional regulation in other systems. It also introduces new methodologies for temporally controlling transcription factor activity, which could lead to refined techniques for investigating gene regulatory networks.

Comments for the author

I'm happy with the current revisions. The authors have addressed key issues, and I feel this is well-suited for publication.

Reviewer 2

Advance summary and potential significance to field

I believe the reagents generated in this study will be broadly useful and have the potential to yield interesting insights. However, the revisions have unfortunately convinced me that the data reported do not justify the conclusions drawn. The paper's central conclusions are undermined by inconsistencies in the data, and the fact that other explanations that are consistent with what is already known were not ruled out.

Comments for the author

The major novel conclusion is that the kinetics of Dorsal nuclear import-export explicitly play a role in determining the expression of target genes. This conclusion is based largely on the observation that snail, a high threshold Dorsal target gene, responds seemingly counterintuitively to two different optogenetic perturbations of Dorsal. To start it's important to understand how the different DL fusions behave in the absence of optogenetic perturbations, which is why all the reviewers asked about this.

© 2024. Published by The Company of Biologists under the terms of the Creative Commons Attribution License (https://creativecommons.org/licenses/by/4.0/).

The authors try to downplay this, but their data clearly shows that even in the dark DL-LEXY and DL-BLID behave very differently (see FigS1). The DL-BLID gradient is both strongly diminished and distorted compared to wild-type Dorsal. DL-BLID levels are almost 10 times lower than DL (Figs S1D & S1E), and the gradient is significantly wider (Fig S1E). In contrast, the gradient encoded by DL-LEXY is essentially the same as wild-type; neither the width nor peak levels being statistically different (Figs S1D & S1E). These differences in the Dorsal gradient are also consistent with the observed phenotypes. Embryos kept in the dark from mothers homozygous for DL-LEXY are “viable and healthy”, while embryos from DL-BLID mothers show severe DV patterning defects, and at room temperature none survive (Irizarry et al., G&D, 2020). Hence even before blue light is shone on DL-LEXY and DL-BLID embryos, DV patterning will be very different because of both direct and indirect effects. This makes it particularly difficult to interpret the observed changes in snail transcription seen when the optogenetic perturbations are performed. This is based on what we already know about the regulation of snail transcription.

It has been well established that in early nc 14 the expression pattern of snail is largely defined by Dorsal and Twist. However later, which is when this study is mostly focused on, it is more complicated and depends on Dorsal indirectly. Early studies where an ectopic Dorsal gradient was introduced into otherwise normal embryos showed that this orthogonal gradient unexpectedly generated gaps in the endogenous snail pattern (Huang et al., G&D, 1997). This was rationalized by invoking Dorsal dependent neurogenic repressors that were cross repressing snail. Data to support this kind of model also appeared in more recent studies that showed that for snail to be transcribed late, there needs to be functional snail protein present (Lagha et al., Cell, 2013). While it is still unclear which gene is responsible for this cross repression, or exactly how it works what is clear is that something more complex is going on, that depends on Dorsal indirectly. So, for example one could easily explain the difference in snail expression in DL-LEXY and DL-BLID via a low/medium threshold neurogenic repressor of snail. Much like sog it would not be expressed in the perturbed DL-BLID embryos, allowing snail to be transcribed. In the DL-LEXY case expression of the repressor would be maintained ensuring snail expression is lost late. Hence indirect effects on snail transcription from a perturbed Dorsal gradient is a significantly more probable explanation than an unknown mechanism that has to do with the kinetics of Dorsal nuclear import-export.

In addition, there were two major inconsistencies. In the first, the authors claim here that both DL-LEXY and DL-BLID support “normal sna expression”, and that “sna expression width is not different in the dark” when you compare DL-LEXY, DL-BLID and wild-type embryos (Fig S1C). This clearly contradicts their previously published work on DL-BLID (Irizarry et al., G&D, 2020), where they state that “dl-BLID embryos kept in the dark were analyzed by FISH and show … a narrower sna expression domain with increased variability at the anterior.”. DL-BLID’s dramatic impact on snail expression is clearly shown in Fig 2 in that paper, where an embryo, kept in the dark, has a sna pattern that is grossly deformed. Snail is completely missing in the anterior half of the embryo, and only a weak sliver of snail remains in the posterior. Since this data is consistent with the observed phenotypes it calls into question the data in this paper claiming that “sna expression width is not different”. Second, in FigS1 the authors clearly show that Dorsal levels are many fold higher in DL-LEXY than in DL-BLID in the dark, which is consistent with the phenotypes. Yet in Figure 4 where DL-LEXY and DL-BLID are quantified live, the starting levels are now almost the same, being within ~10% of each other. Both can’t be true, which calls into question the data presented in Figure 4.

<u>Reviewer 3</u>

Advance summary and potential significance to field

The manuscript is significantly improved. The conclusions stated in the text are now supported by the data provided.

Comments for the author

The writing has also been improved - for example, the abstract is much more informative. The presentation of data - e.g., in Figure 4 - helps the reader and provides a better understanding of the variability in the data.

© 2024. Published by The Company of Biologists under the terms of the Creative Commons Attribution License (https://creativecommons.org/licenses/by/4.0/).

Second revision

Author response to reviewers' comments

******* Reviewer 2 Advance Summary and Potential Significance to Field:**
I believe the reagents generated in this study will be broadly useful and have the potential to yield interesting insights. However, the revisions have unfortunately convinced me that the data reported do not justify the conclusions drawn. The paper's central conclusions are undermined by inconsistencies in the data, and the fact that other explanations that are consistent with what is already known were not ruled out.

Reviewer 2 Comments for the Author:
The major novel conclusion is that the kinetics of Dorsal nuclear import-export explicitly play a role in determining the expression of target genes. This conclusion is based largely on the observation that snail, a high threshold Dorsal target gene, responds seemingly counterintuitively to two different optogenetic perturbations of Dorsal. To start it's important to understand how the different DL fusions behave in the absence of optogenetic perturbations, which is why all the reviewers asked about this.
As written, this suggests that we did not include controls without optogenetic perturbations. However, we hope the reviewer can appreciate that we performed a "dark" control for all experiments.

The authors try to downplay this, but their data clearly shows that even in the dark DL-LEXY and DL-BLID behave very differently (see FigS1). The DL-BLID gradient is both strongly diminished and distorted compared to wild-type Dorsal. DL-BLID levels are almost 10 times lower than DL (Figs S1D & S1E), and the gradient is significantly wider (Fig S1E). In contrast, the gradient encoded by DL-LEXY is essentially the same as wild-type; neither the width nor peak levels being statistically different (Figs S1D & S1E). These differences in the Dorsal gradient are also consistent with the observed phenotypes. Embryos kept in the dark from mothers homozygous for DL-LEXY are "viable and healthy", while embryos from DL-BLID mothers show severe DV patterning defects, and at room temperature none survive (Irizarry et al., G&D, 2020). Hence even before blue light is shone on DL-LEXY and DL-BLID embryos, DV patterning will be very different because of both direct and indirect effects. This makes it particularly difficult to interpret the observed changes in snail transcription seen when the optogenetic perturbations are performed. This is based on what we already know about the regulation of snail transcription.
We didn't intend to "downplay" or mislead the reader but used the comparison to explain why the DL-LEXY fusion is an important advance. We stated in the manuscript "the DL gradient in *dl-LEXY* is more similar to that in control suggesting that the *dl-LEXY* background may be more useful for analysis of dynamic requirements for DL in supporting target gene expression." For Fig S1, the levels in DL-BLID are lower than the control, but the change in width is not that different from dl-LEXY, even if one is statistically significant and the other is not. In addition, the reviewer states "DV patterning will be very different because of both direct and indirect effects" but the expression of *sna* appears similar between all three conditions in Fig S1C. Also, this does not take into account the dark controls shown in Fig 2 and 4.

It is our honest opinion that the fixed data are not as reliable as the live imaging. It is very challenging to control the timing of light application and even embryos kept in the "dark" are inadvertently subjected to some light during the fixation process. We believe the fixed data in Fig S1 have such caveats and are not as easily interpreted as the live imaging since the amount of environmental light exposure is unquantifiable. We included these data as a point of reference and because the reviewer asked for it. For instance even though the DL levels are much lower in the displayed image, the *sna* domain looks similar to DL-LEXY and the control. This suggests that the DL levels may have been reduced more recently (i.e. as embryos were processed and fixed). Thus, the live imaging data is much more controlled and what the basis of this study is focused on.

The reviewer mentions "These differences in the Dorsal gradient are also consistent with the observed phenotypes. Embryos kept in the dark from mothers homozygous for DL-LEXY are "viable

© 2024. Published by The Company of Biologists under the terms of the Creative Commons Attribution License (https://creativecommons.org/licenses/by/4.0/).

and healthy", while embryos from DL-BLID mothers show severe DV patterning defects, and at room temperature none survive (Irizarry et al., G&D, 2020)." We no longer state that DL-LEXY is viable and healthy in this revised manuscript as we have not done a viability assay and cannot make that claim. We would also like to clarify the results of our G&D paper. In that paper, we state that approximately half of the cuticles appear wildtype while half of the cuticles have defects in DL-BLID in the dark. Thus the effects in DL-BLID are not severe, and the effects at room temperature are avoided by doing these experiments at 18°C.
It has been well established that in early nc 14 the expression pattern of snail is largely defined by Dorsal and Twist. However later, which is when this study is mostly focused on, it is more complicated and depends on Dorsal indirectly. Early studies where an ectopic Dorsal gradient was introduced into otherwise normal embryos showed that this orthogonal gradient unexpectedly generated gaps in the endogenous snail pattern (Huang et al., G&D, 1997). This was rationalized by invoking Dorsal dependent neurogenic repressors that were cross repressing snail. Data to support this kind of model also appeared in more recent studies that showed that for snail to be transcribed late, there needs to be functional snail protein present (Lagha et al., Cell, 2013).
The Huang et al. paper that was referenced states that gaps in the *sna* expression were only seen in half of the embryos. This is not a strong set of data on which to base the claim that a neurogenic repressor is responsible. While the authors suggest that a neurogenic repressor could cause this, there is no evidence in this paper that a neurogenic repressor is responsible nor has one been identified in the past 27 years since its publication. Furthermore, our data supports an alternative explanation that DL dynamics, specifically DL being phosphorylated, may affect *sna* transcription and could also explain the results in the Huang et al. study. If phosphorylation affects DL's export rate, and Toll signaling is responsible for the phosphorylation, the gap in *sna* expression in the Huang et al. paper could very well be the same phenomenon we are suggesting occurs in DL-LEXY under blue light.

In addition, the data presented in the Lagha et al. 2013 paper used a *snail* deficiency mutant that removes over 20 genes so a confounding factor could be responsible. Also, while this paper states that late *sna* expression requires Sna protein, late expression is still observed in Fig S5H,J even if it is reduced.

While it is still unclear which gene is responsible for this cross repression, or exactly how it works, what is clear is that something more complex is going on, that depends on Dorsal indirectly. So, for example one could easily explain the difference in snail expression in DL-LEXY and DL-BLID via a low/medium threshold neurogenic repressor of snail. Much like sog it would not be expressed in the perturbed DL-BLID embryos, allowing snail to be transcribed. In the DL-LEXY case expression of the repressor would be maintained ensuring snail expression is lost late. Hence indirect effects on snail transcription from a perturbed Dorsal gradient is a significantly more probable explanation than an unknown mechanism that has to do with the kinetics of Dorsal nuclear import-export.
It is not clear that an indirect effect is the only possible explanation. *sog* is expressed in the DL-BLID embryo. There is a slight ventral shift in the boundary after over 1 hour of blue light illumination. The differences we are comparing in this study in terms of *sna* expression for DL-LEXY versus DL-BLID relate to 20 min illumination schemes; *sog* is not affected when illuminated for only 20 min in late nc14.

In addition, the timescale for this proposed neurogenic repressor X to be activated is on the order of 10-20 min. Loss of *sna-MS2* expression in DL-LEXY under blue light takes ~5 min to completely turn off. In the remaining time of ~15 min, mature *sna* transcripts would need to be degraded, Sna protein would need to be degraded, neurogenic repressor X would need to be transcribed in ventral regions, and then neurogenic repressor X would need to be translated. This requires extremely rapid kinetics that seem improbable. The degradation rate of *sna* transcripts has been estimated as having a halflife of 13.6 min with bounds of 7.2 and 22 min (Boettiger, Cell Reports 2013). This paper also suggests there are ~180 *sna* transcripts/cell. If we use 13.6 min as the half life, this means that at ~14 min there would still be ~90 transcripts/cell. Translation rates have been estimated at 4-35 aa/s (Chen, Nature Cell Biology 2024; Dufourt, Science 2021) with an initiation rate of 13 sec (Dufourt, Science 2021). Sna is 390 aa long, so this would be 24.1-110.5 sec to make a single protein. Thus, Sna protein will continue to be made after *sna* is turned off by removing DL-LEXY from the nucleus with blue light. Pol II rates are estimated at 2.4-3.0 kb/min. Bursts give an average rate of 10s of transcripts per min (J. P. Bothma et al. 2014; Fukaya, Lim, and Levine 2016). If we assume 20 transcripts per minute, it would take 10 min to make 200 transcripts of

© 2024. Published by The Company of Biologists under the terms of the Creative Commons Attribution License (https://creativecommons.org/licenses/by/4.0/).

neurogenic repressor X, and this is assuming Sna is not present to repress its transcription. If we assume the repressor is translated at a similar rate to Sna, this would be 24.1-110.5 sec to translate a single protein. Thus it seems highly unlikely in this period of ~10-15 min that *sna* transcripts would be sufficiently degraded, Sna protein would be sufficiently degraded, and this neurogenic repressor X would be transcribed and translated to meaningful levels. Thus, we think it is more plausible that a direct effect is occurring. We have added a paragraph on the potential of indirect effects, such as a neurogenic repressor X, and why we do not think that occurs to the manuscript.

Furthermore, after 10 min of blue light, DL is able to activate *sna* expression in DL-LEXY when the blue light is removed, suggesting that neurogenic repressor X is not functional at this time (Fig. 2K). In comparison, *sna* expression in DL-BLID begins to recover under blue light, starting at ~7 min of blue light. By the end of the 10 min of blue light, *sna* has recovered close to 50% of the starting levels in DL-BLID (Fig. 2L). During this 10 min period, it is very unlikely for a neurogenic repressor to be derepressed and expressed in DL-LEXY as mentioned in the paragraph above. Thus, it is highly improbable that *sna* is being repressed by a neurogenic gene, and much more likely that the difference between dl-BLID and dl-LEXY is a direct effect of DL on *sna* transcription, which is inactivated by LEXY under blue light (Fig. 2K). Furthermore, we note that the recovery with dl-BLID is likely indirect because Dorsal is degraded and we've shown it's dependent on Twist (Irizarry et al. Genes Dev 2020).

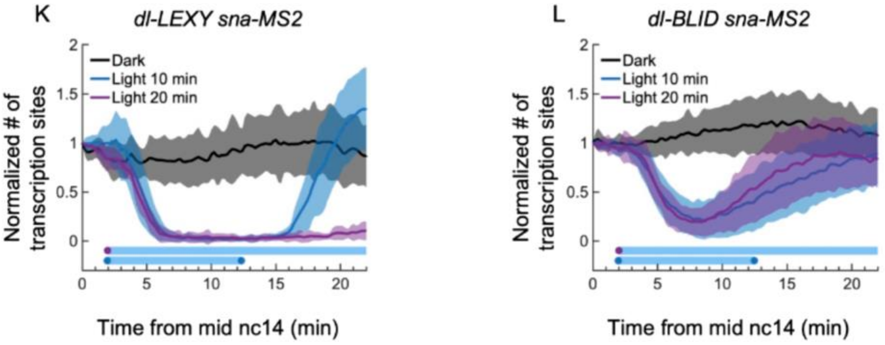

Panels K,L from Figure 2.

In addition, there were two major inconsistencies.
One should not expect the live imaging data and the fixed analysis to be the same. In one case, a powerful laser light is used to illuminate embryos with blue light at a specific time point; with the fixed embryos, a blue box is used to illuminate embryos with a LED light (not strong) and then embryos are processed for fixation in the dark under very low light conditions (but not zero light). However, the trends are consistent. Also, keep in mind that DL-LEXY is able to recover from light treatment, whereas DL-BLID is irreversible. Fixing embryos would exacerbate the difference between DL-LEXY and DL-BLID (i.e. the former able to recover, and the latter not).

In the first, the authors claim here that both DL-LEXY and DL-BLID support "normal sna expression", and that "sna expression width is not different in the dark" when you compare DL-LEXY, DL-BLID and wild-type embryos (Fig S1C). This clearly contradicts their previously published work on DL-BLID (Irizarry et al., G&D, 2020), where they state that "dl-BLID embryos kept in the dark were analyzed by FISH and show … a narrower sna expression domain with increased variability at the anterior.". DL-BLID's dramatic impact on snail expression is clearly shown in Fig 2 in that paper, where an embryo, kept in the dark, has a sna pattern that is grossly deformed. Snail is completely missing in the anterior half of the embryo, and only a weak sliver of snail remains in the posterior. Since this data is consistent with the observed phenotypes it calls into question the data in this paper claiming that "sna expression width is not different".
As we emphasize above, and have made more clear in our paper, it is very challenging to keep embryos completely in the dark and have them undergo fixation. This is why the live imaging work (over 50 movies in this paper) is more dependable than the fixed data. In our opinion, the fixed data are preliminary results that need to be followed up with live imaging. We stand by our results that on average *sna* expression width is not different. The variability in *sna* expression width in the fixed sample is likely due to exposure to light.

© 2024. Published by The Company of Biologists under the terms of the Creative Commons Attribution License (https://creativecommons.org/licenses/by/4.0/).

In the G&D paper, while we noted that *sna* appears narrower, we did not quantify the width of *sna* expression as we have done here.

Second, in FigS1 the authors clearly show that Dorsal levels are many fold higher in DL-LEXY than in DL- BLID in the dark, which is consistent with the phenotypes. Yet in Figure 4, where DL-LEXY and DL-BLID are quantified live, the starting levels are now almost the same, being within ~10% of each other. Both can't be true, which calls into question the data presented in Figure 4.
As stated above, we trust the live imaging data. We think Fig S1 is more likely to have reduced DL due to ambient light exposure, while the live imaging in Fig 4 was kept in the dark or under red light and is thus more representative. We have added a section on the limitations of our study, as well as added sentences stating the potential for ambient light exposure in the fixed data.

Third decision letter

MS ID#: DEVELOP/2024/202775

MS TITLE: Target gene responses differ when transcription factor levels are acutely decreased by nuclear export versus degradation

AUTHORS: James McGehee and Angelike Stathopoulos

I am happy to tell you that your manuscript has been accepted for publication in Development, pending our standard publication integrity checks. Apologies that this took longer than we aim for. I think the revisions you have made strengthen your case, in particular the distinction between live and fixed is important, and this will now be clearer to readers.

Reviewer 3

Advance summary and potential significance to field

I am happy with the response to the reviewer.

Comments for the author

I am happy with the response to the reviewer.

© 2024. Published by The Company of Biologists under the terms of the Creative Commons Attribution License (https://creativecommons.org/licenses/by/4.0/).
